# Uncertainty Quantification of Extratropical Forest Biomass in CMIP5 Models over the Northern Hemisphere

**DOI:** 10.1038/s41598-018-29227-7

**Published:** 2018-07-19

**Authors:** Cheng-En Yang, Jiafu Mao, Forrest M. Hoffman, Daniel M. Ricciuto, Joshua S. Fu, Chris D. Jones, Martin Thurner

**Affiliations:** 1 Department of Civil and Environmental Engineering, University of Tennessee, Knoxville, Tennessee 37996 USA; 2 Climate Change Science Institute and Computational Sciences and Engineering Division, Oak Ridge National Laboratory, Oak Ridge, Tennessee 37831 USA; 3 Climate Change Science Institute and Environmental Sciences Division, Oak Ridge National Laboratory, Oak Ridge, Tennessee 37831 USA; 4 Met Office Hadley Centre, FitzRoy Road, EX1 3PB Exeter, UK; 5 Department of Environmental Science and Analytical Chemistry (ACES), Stockholm University, Svante Arrhenius väg 8, 10691 Stockholm, Sweden; 6 Bolin Centre for Climate Research, Stockholm University, Stockholm, Sweden

## Abstract

Simplified representations of processes influencing forest biomass in Earth system models (ESMs) contribute to large uncertainty in projections. We evaluate forest biomass from eight ESMs outputs archived in the Coupled Model Intercomparison Project Phase 5 (CMIP5) using the biomass data synthesized from radar remote sensing and ground-based observations across northern extratropical latitudes. ESMs exhibit large biases in the forest distribution, forest fraction, and mass of carbon pools that contribute to uncertainty in forest total biomass (biases range from −20 Pg C to 135 Pg C). Forest total biomass is primarily positively correlated with precipitation variations, with surface temperature becoming equally important at higher latitudes, in both simulations and observations. Relatively small differences in forest biomass between the pre-industrial period and the contemporary period indicate uncertainties in forest biomass were introduced in the pre-industrial model equilibration (spin-up), suggesting parametric or structural model differences are a larger source of uncertainty than differences in transient responses. Our findings emphasize the importance of improved (1) models of carbon allocation to biomass compartments, (2) distribution of vegetation types in models, and (3) reproduction of pre-industrial vegetation conditions, in order to reduce the uncertainty in forest biomass simulated by ESMs.

## Introduction

The amount of carbon stored in vegetation biomass in terrestrial ecosystems plays an important role in influencing Earth’s climate. Variations in productivity, respiration, carbon turnover and in carbon allocation to biomass compartments in response to anthropogenic and natural climate change influence the magnitude, spatial distribution and allocation of vegetation carbon stocks. At present, the land biosphere is a carbon sink since atmospheric carbon dioxide (CO_2_) is increasingly sequestered in terrestrial ecosystems^1–5^. The largest pools of terrestrial carbon are within soils and the live biomass of forests^6–9^. Previous studies found that forests presently store 47% of total global carbon on land around the world^3^, and land absorbs approximately one-third of global anthropogenic CO_2_ emissions each year^5^. A recent report showed that 11.5% of total greenhouse gas emissions over the contiguous United States in 2014 were offset by land use, land-use change, and forestry^10^. Therefore, investigations of forest biomass magnitude and carbon allocation are critical to reduce the uncertainty of future global and regional carbon stock estimates in the Earth system models (ESMs).

Differences in parametric and structural representations of vegetation and soil processes in ESMs, as well as uncertainties in simulated climate drivers, result in a wide range of carbon stock estimates. As we will show, biases induced by such parametric and structural differences in the initial state of vegetation biomass (i.e., during the pre-industrial (PI) period following model spin-up) often persist into future biomass predictions. In addition, poor representations of carbon allocation processes^11–13^, inconsistent definitions of biomass in wood and in roots across ESMs^14^, and uncertainties in allocation trade-offs in a changing climate^15,16^ also enlarge uncertainties of projected carbon allocation in the terrestrial biosphere, increasing the range of carbon–climate feedback estimates. Another challenge to minimize uncertainties in ESMs is accurately representing the types and fractional coverage of global forests^17–19^. Despite the creation of several observationally constrained estimates of biomass in above- and below-ground vegetation compartments over the past few decades^3,18,20–24^, the uncertainties of observation-based forest biomass due to different retrieval techniques^25–27^ and upscaling procedures in regions with sparse data availability^28,29^ remain large and poorly quantified. Confidence in the feedback of the vegetation carbon cycle in forest ecosystems to climate change thus requires accurate representation of carbon stocks, forest distribution, and allometric relationships between biomass compartments and the responses of underlying processes to environmental conditions in coupled ESMs.

In this study, we quantify the uncertainty of forest biomass, in terms of its magnitude and carbon allocation among vegetation components, from ESM outputs archived in the Coupled Model Intercomparison Project Phase 5 (CMIP5)^30^ by comparing the simulated results to a recent observation-based data set across the northern extratropical region (30°N–80°N)^24^. Spatial correlations of forest biomass and climatic conditions over this region are also assessed. Some of the ESMs analyzed here allowed for prognostic vegetation distributions using a Dynamic Global Vegetation Model (DGVM) and some did not. In addition, we compare the differences between area-weighted forest biomass at grid cell level and detailed information on forest biomass of individual plant functional types (PFTs) for each model realization. The former provides only the mean carbon density of all PFTs within a grid cell but no carbon density for individual PFTs, while the latter retains different carbon densities for each PFT within a grid cell. Moreover, we analyze the differences of biases in ESMs during the contemporary period and the PI period to investigate the uncertainty caused by parametric or structural model configurations. Hence, taking into account the uncertainty in observations, this study indicates sources of uncertainty for the magnitude and carbon allocation of extratropical forest biomass estimates that may facilitate improved representation of land processes in ESMs that will impact carbon–climate simulations such as those for the Coupled Model Intercomparison Project Phase 6 (CMIP6).

## Results

### Spatial differences of forest fractions and total carbon mass

**Figure 1:**
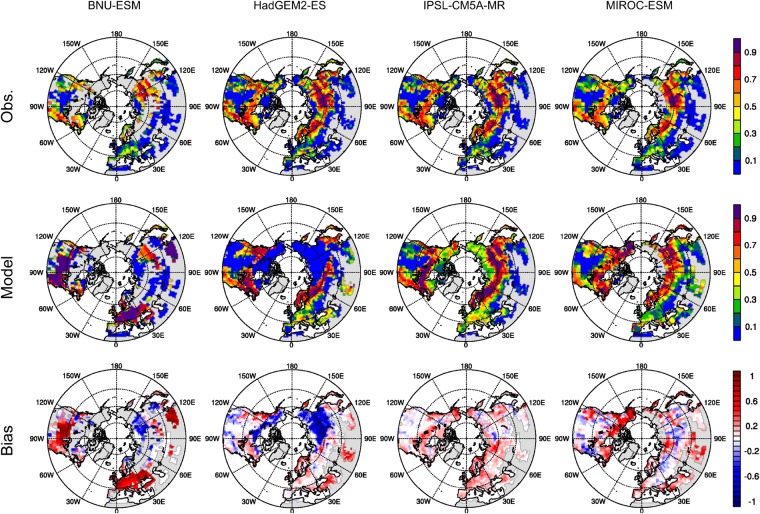
Forest fractions in the northern extratropical regions during the contemporary period for upscaled observations (top row), CMIP5 models (center row), and the differences (bottom row, model–observation). Different resolutions according to model setup are shown (left to right): BNU-ESM, HadGEM2-ES, IPSL-CM5A-MR, and MIROC-ESM. Each model output and its corresponding upscaled observation data are masked by a common-grid land mask. Missing data is presented in light gray color. [Maps were made using the NCAR Command Language v6.4.0 software, 10.5065/D6WD3XH5].

**Figure 2:**
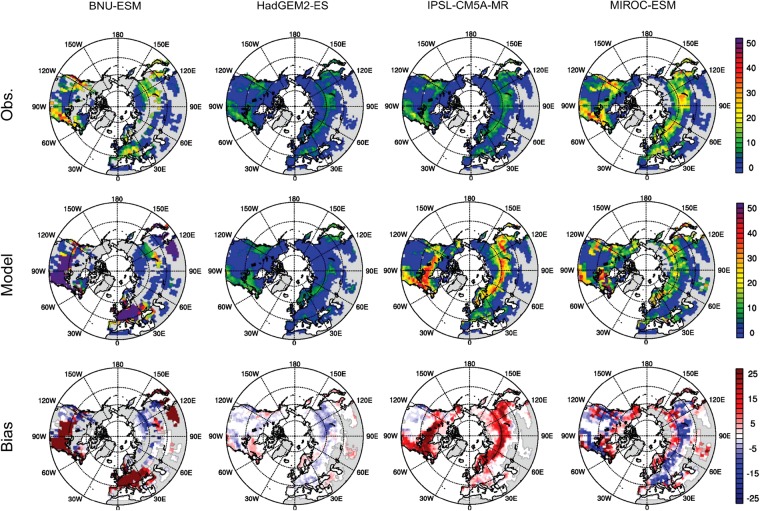
Same as Fig. 1 except for forest total carbon mass (10^−2^ Pg C). [Maps were made using the NCAR Command Language v6.4.0 software, 10.5065/D6WD3XH5].

Four ESMs outputs, one per modeling center, from the eight selected ESM realizations (see Supplementary Table S1) demonstrate substantial differences of spatial forest fractions and forest total biomass compared to observations (Figs 1 and 2). BNU-ESM overestimates forest fractions and total biomass at midlatitudes over Europe, the United States, and China while it underestimates forest fractions in Siberia. These discrepancies of forest fractions (*min* = −0.92; *max* = 0.95) and total carbon mass (*min* = −0.39 Pg C; *max* = 1.63 Pg C) at grid cell level are mainly due to broadleaf deciduous temperate trees in the regions of overestimated forest fractions and C_3_ arctic grass in the regions of underestimated forest fractions (see Supplementary Fig. S1). HadGEM2-ES, one of the two configurations from the Met Office Hadley Centre, noticeably underestimates forest fractions and biomass over Russia and Northern Canada with a magnitude up to 0.98 (*min* = −0.98; *max* = 0.96) and 0.14 Pg C (*min* = −0.14 Pg C; *max* = 0.14 Pg C), respectively. The uncertainty originates from the model identifying these regions as shrub-dominant (see Supplementary Fig. S1) whereas large portions of needleleaf forest are present in the observations. IPSL-CM5A-MR, one of the three ESM configurations from the Institut Pierre Simon Laplace (IPSL), shows biases in forest total biomass ranging from −0.19 Pg C to 0.41 Pg C with relatively lower biases in forest fractions (*min* = −0.58; *max* = 0.78) compared to the other three centers’ ESMs; nevertheless, they generally exhibit positive biases in forest fractions across northern high latitudes, especially between 45°N–60°N. This is a consequence of excessively large fractions of boreal needleleaf evergreen trees and boreal needleleaf summergreen trees apportioned in the three IPSL ESMs (see Supplementary Fig. S1). MIROC-ESM, one of the two model configurations from the Model for Interdisciplinary Research on Climate (MIROC), demonstrates that the simulated forest fractions deviate from the observations by −0.68 to 0.97 with overpredicted forest fractions in Northern Canada and Eurasia north of 60°N (boreal forests) but with underestimated forest fractions over the northeast coast of North America (dominated by C_4_ grasses) and over Eurasia south of 60°N (dominated by crops) (see Supplementary Fig. S1). The differences of global forest total biomass range from −0.37 Pg C to 0.62 Pg C. Spatial differences of forest fractions and total biomass of all eight ESMs are illustrated in Supplementary Figs S2 and S3.

Differences between the observed and modeled forest area also increase the uncertainty of forest biomass estimates when converting the carbon density retrieved from the CMIP5 archive and from the observations to carbon mass. We apply a common-grid land mask (see Methods) to each grid cell for each ESM output and its corresponding upscaled observations (obsESMs) based on the Global Land Cover 2000 (GLC2000) data^31^. Different ESMs have different masks depending on the forest fraction and biomass availability in grid cells for an ESM and its obsESM. Masked forest area (“effective forest area” hereafter) and thus forest total biomass can exhibit substantial variations within ESMs and within obsESMs. For example, smaller effective forest area is found in the upscaled observation for the BNU-ESM (obsBNU-ESM) compared to that for the other obsESMs. This is because the mask applied to obsBNU-ESM excludes grid cells where BNU-ESM considers little or no forest existence in the model. With the common-grid land mask applied, the effective forest area for ESMs is (1.2–2.0) × 10^9^ ha, whereas that for obsESMs is (0.8–1.6) × 10^9^ ha (see Supplementary Fig. S4). Modeled effective forest area is underestimated in both HadGEM2 ESMs (−24% for HadGEM2-CC and −19% for HadGEM2-ES) while overestimated effective forest area is found in BNU-ESM (47%) as well as in the IPSL ESMs and the MIROC ESMs (29–36%). This uncertainty is mainly attributable to the lack of the ESMs representing the observed land cover types shown in GLC2000. For instance, BNU-ESM allocates C_3_ arctic grass rather than any forest type in most regions north of 50°N. Another example can be found from the HadGEM2 ESMs in which the majority of land over Asian Russia and northwest North America is covered by shrubs, resulting in low proportions of forest types being allocated in the models. Due to large uncertainties between ESMs and obsESMs caused by different land cover definitions, we apply various thresholds of the forest fraction (*F*_*f*_) to examine how uncertainty varies with *F*_*f*_. It is expected that higher *F*_*f*_ levels produce grid cells more dominated by homogeneous forest types and thus minimize the inconsistencies of PFT definitions between ESMs and obsESMs. However, higher *F*_*f*_ thresholds result in less data availability. For example, by increasing *F*_*f*_ from 0 to 0.1, the effective forest area significantly reduces to two-thirds of its value at *F*_*f*_ = 0 for BNU-ESM and to 83–88% for the other ESMs (see Supplementary Fig. S4). Therefore, this study applies *F*_*f*_ = 0 to avoid utilizing forest biomass data from only a few grid cells to represent all extratropical forest biomass over the Northern Hemisphere.

**Figure 3:**
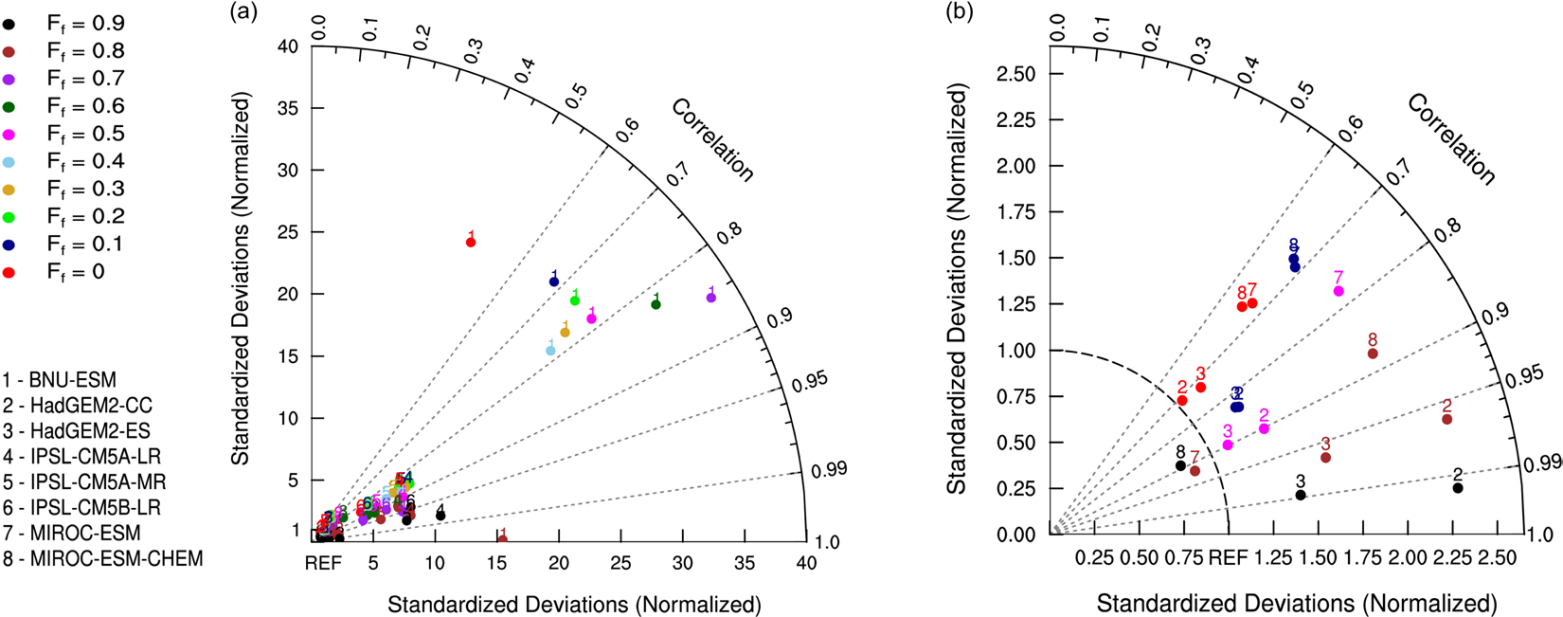
Taylor diagrams of (**a**) all model results and (**b**) model results of standardized deviations smaller than 2.5 for the northern extratropical forest carbon mass at various forest fraction thresholds (*F*_*f*_) ranging from 0.0 to 0.9. Model numbers represent the CMIP5 models including (1) BNU-ESM, (2) HadGEM2-CC, (3) HadGEM2-ES, (4) IPSL-CM5A-LR, (5) IPSL-CM5A-MR, (6) IPSL-CM5B-LR, (7) MIROC-ESM, and (8) MIROC-ESM-CHEM. [Maps were made using the NCAR Command Language v6.4.0 software, 10.5065/D6WD3XH5].

A further investigation on the similarity of the spatial patterns of forest biomass between ESMs and obsESMs is illustrated by Taylor diagrams (Fig. 3a,b). The ESMs, while able to capture the spatial distributions of forest biomass at high *F*_*f*_ levels, have huge variations in terms of carbon mass magnitude owing to different land cover types and forest fractions (Fig. 3a). Correlations between the spatial patterns of simulated and observed forest carbon mass increase with *F*_*f*_ for all ESMs, while the variances of modeled forest biomass are generally greater than that of observed values since the normalized deviations are greater than one (except for MIROC-ESM at *F*_*f*_ = 0.8 and MIROC-ESM-CHEM at *F*_*f*_ = 0.9) (Fig. 3b). BNU-ESM exhibits the lowest skill scores (see Eq. (1) in Methods) (0.006 ± 0.005) because it has the largest variance among all ESMs at all *F*_*f*_ levels (Fig. 3a; Supplementary Fig. S5). The IPSL ESMs perform slightly better than BNU-ESM, but their large variances cause low skill scores (0.11 ± 0.04) as well (Fig. 3a; Supplementary Fig. S5). Higher skill scores are found for the HadGEM2 ESMs (0.85 ± 0.10) and the MIROC ESMs (0.64 ± 0.14) (Fig. 3b; Supplementary Fig. S5).

### Magnitude of forest biomass uncertainty

**Figure 4:**
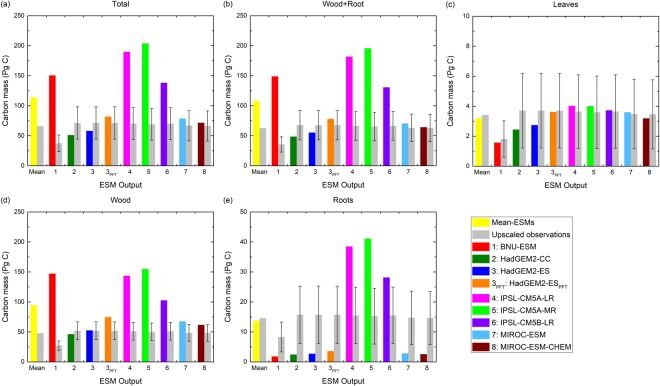
Global extratropical forest total carbon mass (Pg C) in the Northern Hemisphere for individual forest compartments: (**a**) all compartments, (**b**) wood and roots, (**c**) leaves, (**d**) wood, and (**e**) roots during the contemporary period at *F*_*f*_ = 0. Columns in light gray are the masked observations at each model resolution with error bars showing the uncertainty. The colored columns represent modeled results from BNU-ESM (1, red), HadGEM2-CC (2, green), HadGEM2-ES (3, blue), HadGEM2-ES with detailed PFT information (3_PFT_, orange), IPSL-CM5A-LR (4, magenta), IPSL-CM5A-MR (5, light green), IPSL-CM5B-LR (6, violet), MIROC-ESM (7, light blue), and MIROC-ESM-CHEM (8, dark brown). The mean value (yellow) is the average of all model results. (**a**,**b**) and (**d**) use the same vertical scale while (**c**) and (**e**) use different vertical scales. [The figure was made using the Origin software (OriginLab, Northampton, MA, USA)].

The biases of masked global forest total carbon mass in each biomass compartment, including the lumped total biomass from all forest compartments and the biomass in leaves, in wood, and in roots, are investigated along with the combined wood and roots (“Wood + Root”) to avoid inconsistent definitions of root biomass between ESMs^14^. Results demonstrate that the biases of modeled global forest total carbon mass at *F*_*f*_ = 0 ranges from −20.3 Pg C in HadGEM2-CC to 134.7 Pg C in IPSL-CM5A-MR (Fig. 4a). In terms of individual forest compartments, the combined Wood + Root biomass has biases ranging from −19.0 Pg C in HadGEM2-CC to 130.4 Pg C in IPSL-CM5A-MR (Fig. 4b); the biases of biomass in leaves ranges from −1.3 Pg C in HadGEM2-CC to 0.4 Pg C in IPSL-CM5A-MR (Fig. 4c); the biases of biomass in wood ranges from −5.7 Pg C in HadGEM2-CC to 120.0 Pg C in BNU-ESM (Fig. 4d); the biases of biomass in roots ranges from −13.3 Pg C in HadGEM2-CC to 25.8 Pg C in IPSL-CM5A-MR (Fig. 4e). A further examination on utilizing detailed biomass data at grid cell level rather than the grid area-weighted level from the HadGEM2-ES model results illustrates relatively smaller biases, though the bias of forest total biomass changes from negative (−13.4 Pg C) by utilizing grid area-averaged biomass data to positive (10.5 Pg C) by utilizing detailed biomass data at the grid cell level (see Supplementary Table S2). Compared to the mean value of forest total biomass from all obsESMs, that from all ESMs is overpredicted by 47.8 Pg C; for individual forest compartments, the mean value from all ESMs shows overpredicted forest biomass in Wood + Root by 43.4 Pg C, in wood by 41.5 Pg C, and in roots by 1.9 Pg C, while the biomass in leaves is underpredicted by 0.2 Pg C. Note that all ESMs predict the biomass in leaves within the uncertainty range of obsESMs, while BNU-ESM and the IPSL ESMs predict too much forest total biomass and biomass in Wood + Root due to overestimated biomass in wood (Fig. 4d). The institutional mean values of forest biomass for each modeling center show enlarged magnitude of bias in total mass (7.7 Pg C) and biomass in Wood + Root (10.2 Pg C) compared to multi-model mean values. The increased bias of biomass in wood (14.8 Pg C) is the major cause of the increasing discrepancies, even though the institutional mean biomass in roots is reduced (−4.57 Pg C). Details of extratropical forest carbon mass across 30° N–80° N for each ESM and for each modeling center is shown in Supplementary Table S2.

**Figure 5:**
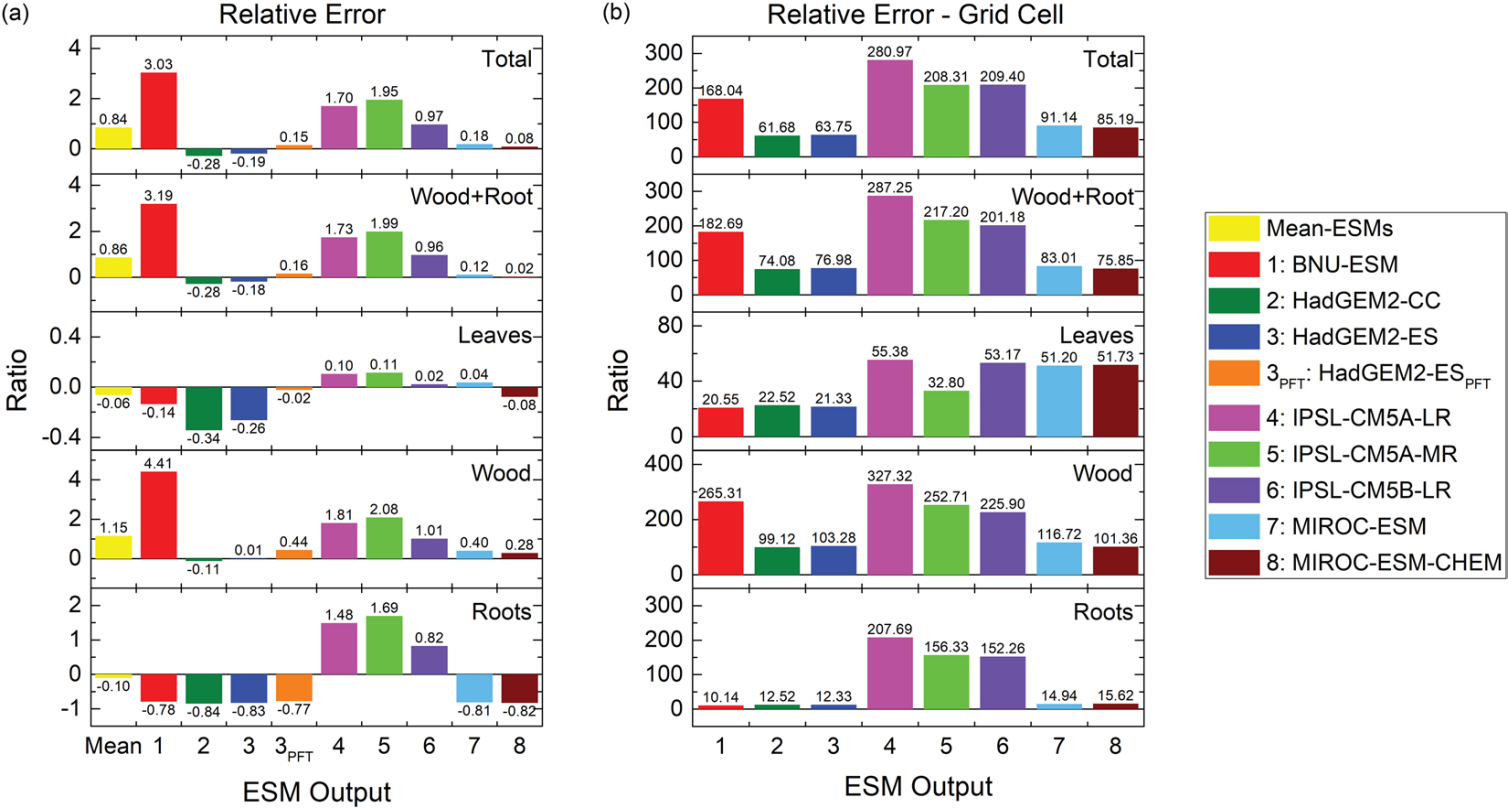
Relative errors for (**a**) global forest total carbon mass and (**b**) averaged forest total carbon mass at grid cell level during the contemporary period. Each sub-panel (top to bottom) represents all compartments (Total), wood and roots (Wood + Root), leaves, wood, and roots at *F*_*f*_ = 0. Model numbers are the same as used in Fig. 4. [The figure was made using the Origin software (OriginLab, Northampton, MA, USA)].

According to the masked forest biomass in ESMs and obsESMs, the magnitude of biases varies over a wide range. To minimize the influences of various masks applied to ESMs, we evaluate the model uncertainty in terms of relative errors (*ER*, see Eq. (2) in Methods) so that underestimates or overestimates in model results can be quantified in a standardized way. The multi-model *ER* average from the *ER*s of all ESMs shows that ESMs simulate excess forest total biomass (*ER*_*total*_ = 0.84), biomass in wood (*ER*_*wood*_ = 1.15), and biomass in Wood + Root (*ER*_*wood+root*_ = 0.86), whereas underestimated biomass in leaves (*ER*_*leaf*_ = −0.06) and biomass in roots (*ER*_*root*_ = −0.10) are found at *F*_*f*_ = 0 (Fig. 5a; institutional-averaged results are shown in Supplementary Fig. 6). The overestimation of global forest total carbon mass is mainly a result of overestimated biomass in wood. All IPSL ESMs significantly overestimate forest biomass in all compartments (*ER*_*total*_ = 0.97–1.95, *ER*_*leaf*_ = 0.02–0.11, *ER*_*wood*_ = 1.01–2.08, *ER*_*root*_ = 0.82–1.69, and *ER*_*wood*+*root*_ = 0.96–1.99), while HadGEM2-CC simulates too little forest biomass in all compartments (*ER*_*total*_ = −0.28, *ER*_*leaf*_ = −0.34, *ER*_*wood*_ = −0.11, *ER*_*root*_ = −0.84, and *ER*_*wood*+*root*_ = −0.28). BNU-ESM exhibits the largest overestimates in forest total carbon amount by more than three times the observational estimate (*ER*_*total*_ = 3.03) due to extremely overestimated biomass mass in wood (*ER*_*wood*_ = 4.41). For individual forest compartments, MIROC-ESM-CHEM has the smallest deviation from observations for forest total biomass (*ER*_*total*_ = 0.08) and for biomass in Wood + Root (*ER*_*wood*+*root*_ = 0.02); IPSL-CM5B-LR shows the smallest deviation for biomass in leaves (*ER*_*leaf*_ = 0.02); HadGEM2-ES shows the smallest deviation for biomass in wood (*ER*_*wood*_ = 0.01); BNU-ESM shows the smallest deviation for biomass in roots (*ER*_*root*_ = 0.04). Similar to the magnitude of forest carbon mass, utilizing detailed PFT-level biomass data from HadGEM2-ES results in better model performance with smaller absolute *ER* magnitude (except for biomass in wood) compared to using grid area-weighted biomass products (Fig. 5a). The *ER* value changes from −0.19 to 0.15 for forest total biomass, from −0.18 to 0.16 for biomass in Wood + Root, from −0.26 to −0.02 for biomass in leaves, from 0.01 to 0.44 for biomass in wood, and from −0.83 to −0.77 for biomass in roots.

All ESMs exhibit positive global-averaged relative errors at grid cell level (*ER*_*grid*_, see Eq. (3) in Methods) in all compartments at *F*_*f*_ = 0 (Fig. 5b; institutional-averaged results are shown in Supplementary Fig. 6), indicating all ESMs overestimate forest biomass. The magnitude of absolute *ER*_*grid*_ values can be two to three orders of magnitude larger than *ER*. The largest relative errors for forest total biomass (*ER*_*grid*,*total*_) and for individual forest compartments (*ER*_*grid*,*leaf*_, *ER*_*grid*,*wood*_, *ER*_*grid*,*root*_, and *ER*_*grid*,*wood*+*root*_) at grid cell level are found in IPSL-CM5A-LR (*ER*_*grid*,*total*_ = 281.0, *ER*_*grid*,*leaf*_ = 55.4, *ER*_*grid*,*wood*_ = 327.3, *ER*_*grid*,*root*_ = 207.7, *ER*_*grid*,*wood*+*root*_ = 287.3). BNU-ESM has the smallest relative error for biomass in leaves (*ER*_*grid*,*leaf*_ = 20.6) and in roots (*ER*_*grid*,*root*_ = 10.1), whereas HadGEM2-CC has the smallest relative errors for forest total biomass (*ER*_*grid*,*total*_ = 61.68) and biomass in wood (*ER*_*grid*,*wood*_ = 99.12) and in Wood + Root (*ER*_*grid*,*wood*+*root*_ = 74.08) at grid cell level. These huge relative errors at grid cell level imply that ESMs exhibit substantial overestimates at certain grid cells in which observed forest biomass is very small and thus large variations of forest biomass at grid cell level are expected for all ESMs (see Supplementary Table S3).

### Carbon allocation to forest compartments

**Figure 6:**
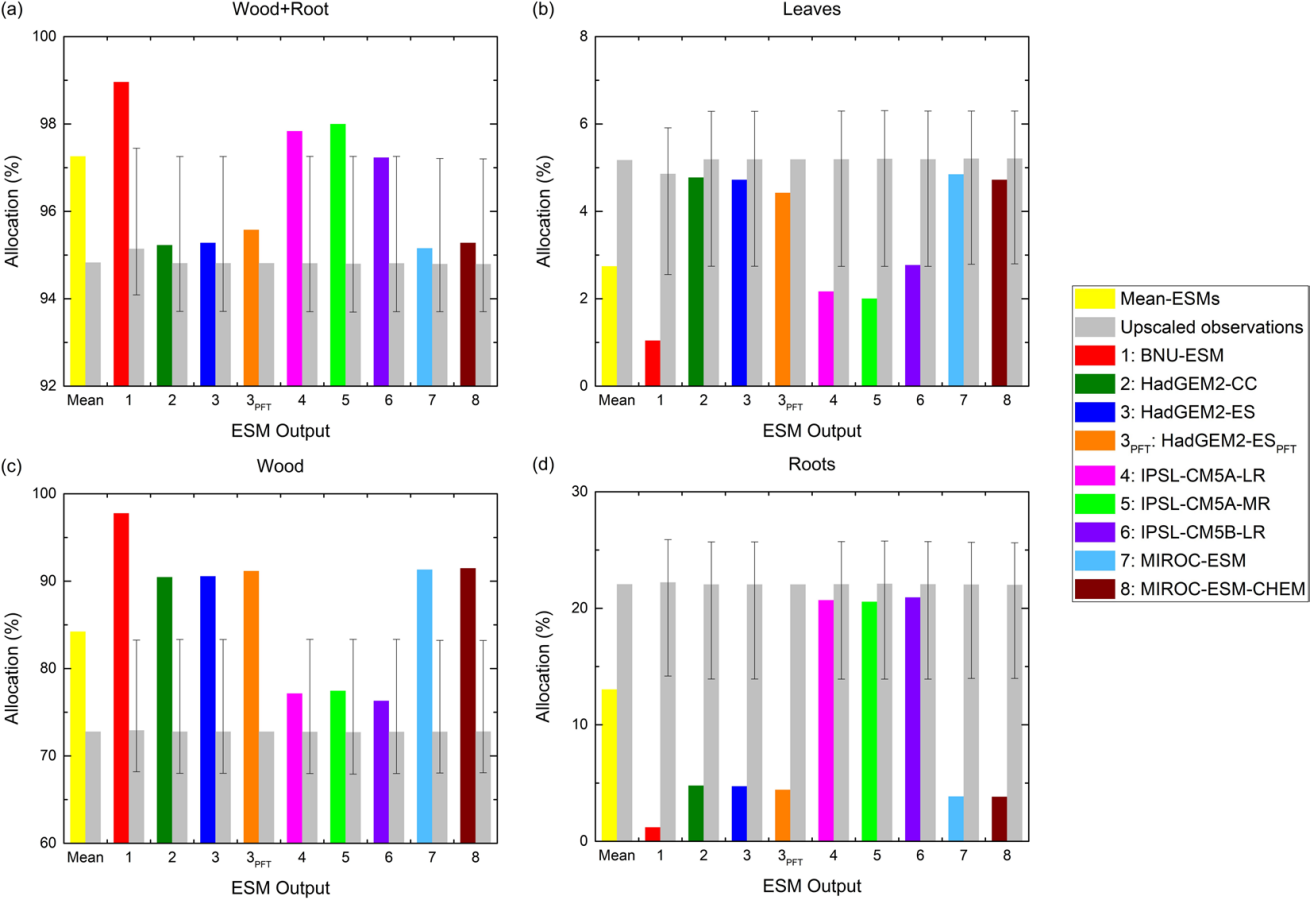
Allocations of global forest total carbon mass in (**a**) wood and roots, (**b**) leaves, (**c**) wood, and (**d**) roots during the contemporary period at *F*_*f*_ = 0. Colors are the same as used in Fig. 4. (**a** and **c**) use the same vertical scale while (**b**) and (**d**) use different vertical scales. [The figure was made using the Origin software (OriginLab, Northampton, MA, USA)].

Global carbon mass allocated in forest biomass compartments for all but the IPSL ESMs is consistently over-apportioned to wood but under-apportioned to leaves and roots (Fig. 6). The carbon allocation to forest compartments for all obsESMs ranges from 93.7–97.4% in Wood + Root, 2.6–6.3% in leaves, 67.9–83.3% in wood, and 13.9–25.9% in roots, while the mean values of all obsESMs with one standard deviation are 96.5% ± 1.5%, 3.5% ± 1.5%, 87.1% ± 7.9%, and 9.4% ± 8.5%, respectively. All IPSL ESMs apportion carbon mass to wood and to roots, with an institutional average of 76.96% ± 0.59% for the former and 20.73% ± 0.19% for the latter, the closest to the observed values. The institutional averages from the two MIROC ESMs and from the two HadGEM2 ESMs are found to allocate the carbon mass in Wood + Root (95.25% ± 0.04% for HadGEM2 ESMs and 95.22% ± 0.09% for MIROC ESMs) and in leaves (4.75% ± 0.04% for HadGEM2 ESMs and 4.78% ± 0.09% for MIROC ESMs) the closest to the observations. These results suggest that the HadGEM2 ESMs and the MIROC ESMs combine the biomass of coarse roots into the woody biomass pool, so that too much carbon is apportioned to wood and too little is apportioned to roots; on the contrary, the IPSL ESMs likely define the carbon mass in roots as the sum of coarse and fine root carbon mass since the carbon allocations are closer to the observed values.

### Spatial covariation of forest total biomass and climatic conditions

**Figure 7:**
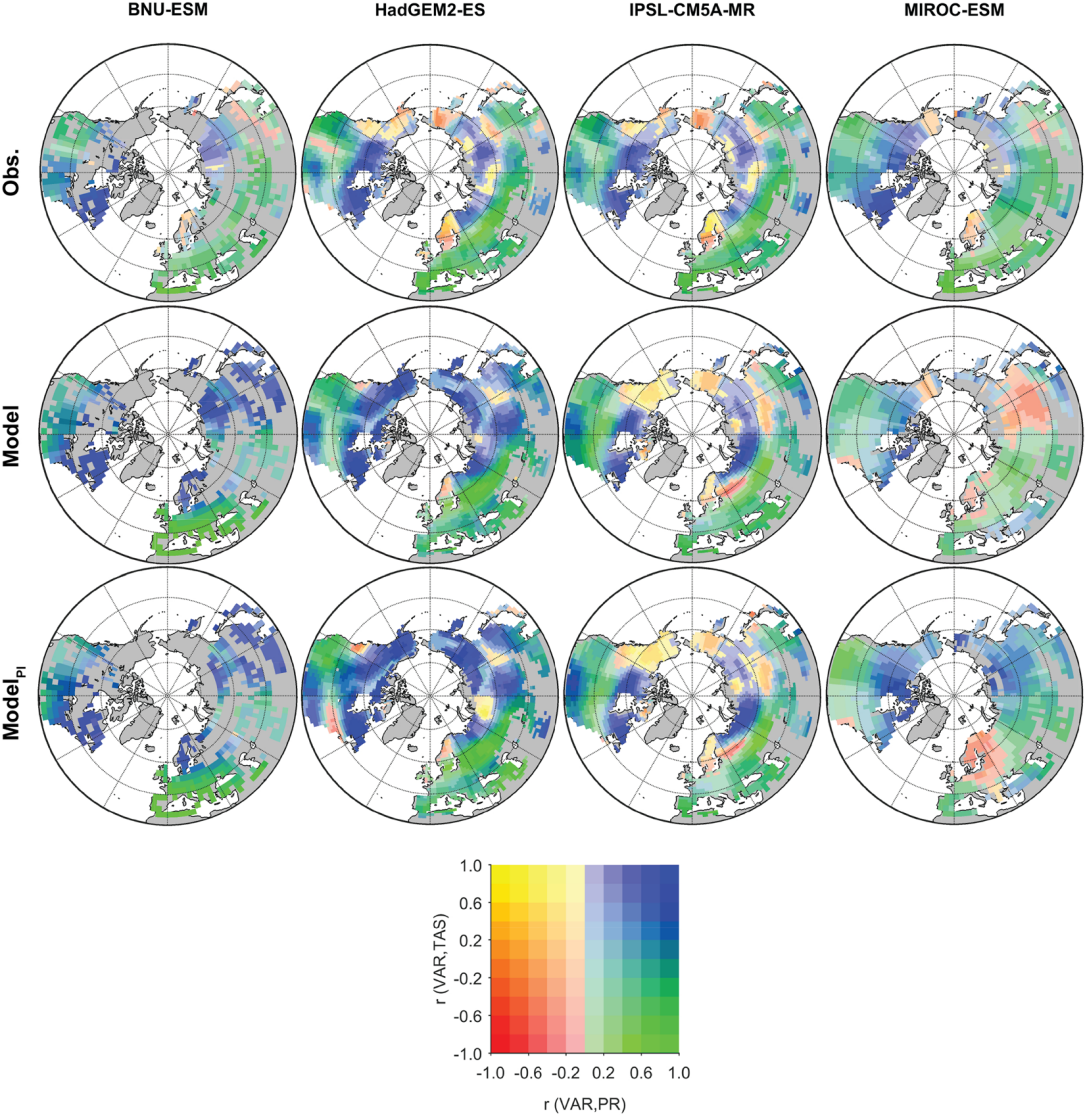
Global correlation maps of observation (top row), model outputs during the contemporary period (middle row), and model outputs at pre-industrial period (bottom row) for each model resolution. Missing data on land is shown in gray color. The color table represents the magnitude of correlations between forest total carbon mass and precipitation (horizontal) and between forest total carbon mass and surface temperature (vertical). Grid resolutions include (left to right): BNU-ESM, HadGEM2-ES, IPSL-CM5A-MR, and MIROC-ESM. [Maps were made using Matlab version 2016a, https://www.mathworks.com/products/matlab.html].

The spatial correlations of forest total biomass and climatic conditions are generally consistent in ESMs and obsESMs in terms of the high positive correlations between biomass and precipitation (PR) at midlatitudes (30°N–60°N), while surface temperature (TAS) becomes as important as PR at high latitudes (60°N–75°N) (Fig. 7). Due to the data aggregation process at different grid resolutions, some minor discrepancies exist between obsESMs, even the ones from the same modeling center (see Supplementary Fig. S7). The three major inconsistent signals between ESMs and obsESMs are located in Alaska, northern China, and Scandinavia. In Alaska, the observations show positive correlations of forest total biomass with only TAS while both HadGEM2 ESMs show positive correlations of forest total biomass with both PR and TAS; IPSL-CM5A-MR and the MIROC ESMs capture the responses of forest total biomass to PR and TAS relatively better than the other ESMs. In northern China, models with higher resolutions (HadGEM2 ESMs and IPSL-CM5A-MR) exhibit weak positive correlations between forest total biomass and TAS, whereas the variations of forest total biomass are weakly negatively correlated with PR. These signals are not seen in either observations or ESMs at lower resolutions. In Scandinavia, the observed forest carbon mass at different model resolutions shows negative forest biomass responses to both PR and TAS changes. All IPSL and MIROC ESMs demonstrate similar results except that MIROC-ESM-CHEM shows a positive correlation between observed forest total biomass and PR in western Russia, while a negative correlation is found in the model (see Supplementary Fig. S7). In contrast, forest total biomass is positively correlated to changes of PR and TAS in BNU-ESM and the HadGEM2 ESMs. Overall, the correlations of spatial variations in forest total biomass to that in PR and in TAS for IPSL-CM5A-MR are closer to the observations. A further analysis of the forest biomass in climatic PR–TAS space on a Whittaker diagram^32^ highlights the spatial distributions of forest biomes in ESMs as compared with obsESMs. For this analysis, we combined all model forest PFTs into four biome categories—broadleaf evergreen tree, needleleaf evergreen tree, broadleaf deciduous tree, and needleleaf deciduous tree—except for the HadGEM2 ESMs, which were grouped into broadleaf tree and needleleaf tree only because of its simplified PFT representation (see Supplementary Tables S4 and S5). We found that ESMs roughly capture the spread of forest biomes in the Northern Hemisphere extratropics, except that ESMs have very few grid cells representing forests below −10 °C (see Supplementary Fig. S8). The masked observational biomass in the modeled PR–TAS space, compared to that in the observed PR–TAS space, also shows fewer grid cells representing forests below −15 °C implying biases in modeled climatic conditions. Among the ESMs, only BNU-ESM shows broadleaf deciduous forests spanning a wide range in TAS, from −10 °C to 20 °C, while the observations indicate a narrower range for these forests.

### Spin-up impacts

**Figure 8:**
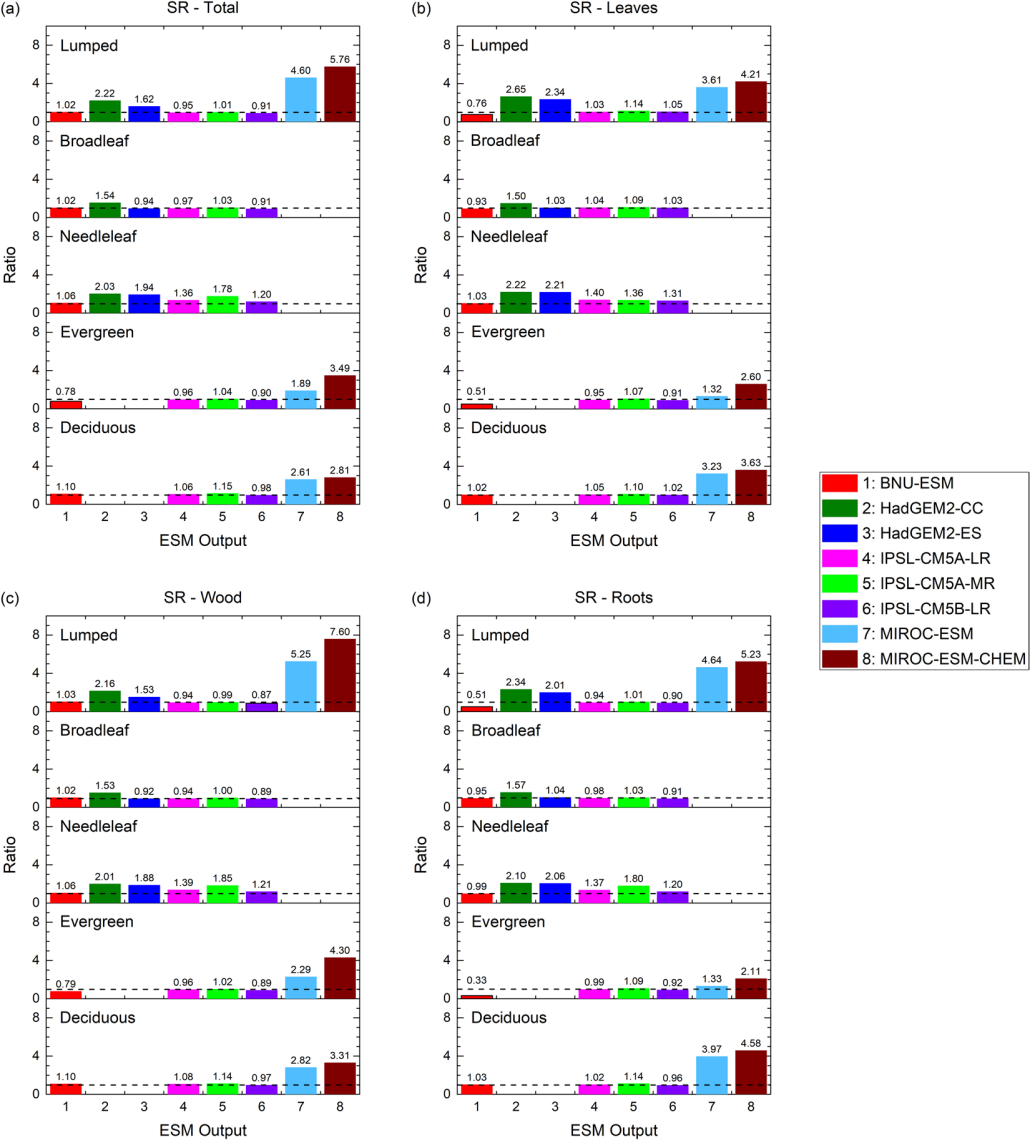
Ratios for global mean relative errors of masked carbon mass at grid cell level during the pre-industrial period to that during the contemporary period at *F*_*f*_ = 0. Forest compartments include (**a**) all compartments, (**b**) leaves, (**c**) wood, and (**d**) roots. The colors and model numbers are the same as used in Fig. 4. Forest types in each sub-panel (top to bottom): lumped, broadleaf, needleleaf, evergreen, and deciduous trees. Dashed lines represent *SR* = 1. [The figure was made using the Origin software (OriginLab, Northampton, MA, USA)].

The ratios of *ER*_*grid*_ for four forest types (see Supplementary Table S6) during the PI period over that during the contemporary period are calculated as an index (*SR*, see Eq. (4) in Methods) for evaluating the uncertainty caused by the spin-up processes. For individual forest compartments at *F*_*f*_ = 0, BNU-ESM shows that *SR* values are close to unity in forest total biomass (*SR*_*total*_ = 1.02) and biomass in leaves (*SR*_*leaf*_ = 0.76), in wood (*SR*_*wood*_ = 1.03), and in roots (*SR*_*root*_ = 0.51). Similar to BNU-ESM, the mean *SR* values of all forest compartments simulated by the IPSL ESMs are also close to unity (*SR*_*total*_ = 0.95 ± 0.05, *SR*_*leaf*_ = 1.08 ± 0.06, *SR*_*wood*_* = *0.94 ± 0.06, *SR*_*root*_ = 0.95 ± 0.05) (Fig. 8). These results imply the uncertainty may have been introduced at the beginning of the simulations in the ESMs due to the initial conditions derived from spin-up output. Similar unity *SR* values are also found in individual forest types, especially for the broadleaf and for the deciduous forests for the ESMs from these two modeling centers. On the contrary, moderate to substantial differences of *ER*_*grid*_ values for the HadGEM2 ESMs (*SR*_*total*_ = 1.92 ± 0.43, *SR*_*leaf*_ = 2.49 ± 0.21, *SR*_*wood*_* = *1.85 ± 0.45, *SR*_*root*_ = 2.17 ± 0.23) and the MIROC ESMs (*SR*_*total*_ = 5.18 ± 0.82, *SR*_*leaf*_ = 3.91 ± 0.43, *SR*_*wood*_* = *6.42 ± 1.66, *SR*_*root*_ = 4.94 ± 0.42), especially MIROC-ESM-CHEM (*SR*_*total*_ = 5.76, *SR*_*leaf*_ = 4.21, *SR*_*wood*_ = 7.60, *SR*_*root*_ = 5.23), suggest uncertainty carried from the spin-up outputs due to vegetation parameterizations are minimized in these ESMs.

## Discussion

The present study evaluates the uncertainty of forest biomass across northern extratropical latitudes from eight coupled ESM simulations. Instead of regridding all ESM outputs and a recent observation-based data to a common grid resolution that induces additional uncertainty, we aggregate the fine-resolution observational data to the same grid resolution of each ESM so that each ESM output can be compared fairly to the aggregated observations. We examine the sources of uncertainty of forest biomass estimates through the forest fractions, the magnitude of carbon mass differences, the carbon mass allocations in forest compartments, and the biases carried from the spin-up results. Inconsistent definitions of PFTs between ESMs and observations contribute to the uncertainty of forest spatial distributions and thus large variations of forest fractions and biomass in each grid cell are found in ESMs^33^. After masks are applied, such inconsistencies cause significant differences in effective forest area among ESMs and among obsESMs. We also find that the unmasked total carbon mass in each aggregated observation data set for different ESM grid resolutions is the same but becomes smaller after forest-only masks are applied to grid cells (see Supplementary Fig. S9). The magnitude of discrepancy in modeled forest fraction and total biomass can be up to 0.98 and up to 1.63 Pg C, respectively, compared to the aggregated observations at the grid cell level. The bias of masked forest total biomass estimates in the northern extratropical region ranges from −20.3 Pg C to 134.7 Pg C and is mainly attributed to the bias in the wood compartment (−5.7 Pg C to 120.0 Pg C). In addition, CMIP5 models typically do not output the varying carbon density for all available PFTs within a grid cell. Some of the forest biomass uncertainty results from aggregation of PFT-level carbon pools to the grid cell level for purposes of reporting. This causes the loss of carbon density heterogeneity at the grid cell level and tends to lead to larger biases in carbon mass estimates. Analysis of HadGEM2-ES outputs with detailed PFT-level carbon pools demonstrates a reduction in modeled forest total carbon mass bias of 2.9 Pg C compared to the grid cell-level data reported in the CMIP5 archive. Hence, we suggest adding detailed PFT-level output of carbon stock and flux variables in CMIP6 simulations so that the uncertainty in future carbon mass estimates reported to the Sixth Assessment Report of the Intergovernmental Panel on Climate Change can be attributed to ecosystem processes for each PFT. While causes of individual variations among model estimates of biomass are difficult to ascertain, multiple sources of uncertainty, as described below, contribute, including prognostic vegetation dynamics, land use and land cover change, and carbon allocation.

For the uncertainty of forest total carbon mass, our analyses show that four of the ESM simulations, if observational uncertainty is considered, overestimate the total carbon mass mainly due to overestimated biomass in wood. Since all ESMs consider carbon cycle feedbacks and land use change, the overestimates are possibly associated with biases in PFT structures and parameters in the DGVM and the land cover change setup in the ESMs. Traditionally, climate models use a prescribed coverage of land surface types, based on present-day observed land cover, but increasingly, ESMs are simulating the vegetation coverage internally using process-based DGVMs embedded within their land-surface scheme. This means that the models may incur biases and errors in the coverage of vegetated lands (e.g. trees, grasses, etc.), but such DGVMs offer the advantage that changes in vegetation cover in response to climate and CO_2_ can be simulated interactively. It has been shown that future changes in land cover induced by climate change can be just as big as changes induced by anthropogenic land-use change, and so such schemes are vital to fully capture changes in the terrestrial carbon cycle^34^. In our results, BNU-ESM used a DGVM to provide prognostic, climate-driven land cover change, but did not consider anthropogenic land use change; the three IPSL ESMs simulated the forest biomass with prescribed land use change but without prognostic land cover change; the two MIROC ESMs and the two HadGEM2 ESMs employed DGVMs for prognostic land cover change and incorporated prescribed land use change impacts (see Supplementary Table S1). Our analyses show that BNU-ESM and the IPSL ESMs exhibit large uncertainty in forest biomass estimates (institutional mean *ER*_*total*_ = 3.03 and 1.54, respectively), while relatively smaller biases are found in the HadGEM2 ESMs and in the MIROC ESMs (institutional mean *ER*_*total*_ = −0.11 and 0.13, respectively). Furthermore, Northern Hemisphere extratropical forest distributions in Whittaker climate space are mostly consistent with aggregated observations, except that BNU-ESM shows broadleaf deciduous trees below −10 °C, where needleleaf deciduous trees are found in the observations (see Supplementary Fig. S8). According to the Whittaker diagrams, vegetation models from all modeling centers, except BNU-ESM, capture biome distributions reasonably well; however, too little forest biomass was simulated by ESMs in low temperature zones, i.e., needleleaf or needleleaf deciduous trees were underrepresented in the ESMs. For BNU-ESM, forest biomass was underestimated over northern Asia because too much carbon was allocated to C_3_ arctic grass instead of to needleleaf forests. Biases in modeled climatic conditions also induce uncertainty in forest biomass estimates. The Whittaker diagrams of observed biomass in modeled climate space show a bias towards warmer temperatures over land in regions where needleleaf deciduous trees are located in the observations. These results suggest that (1) better PFT representations in the DGVM, (2) land use change, and (3) reducing biases in climatic conditions that affect plant productivity should all be considered in simulations to reduce the uncertainty of future forest carbon pool estimates. Further simulations for each model can help improve the understanding of the impact on biomass uncertainty due individually to the DGVM, land use changes, and climatic conditions.

In addition to the DGVM and land cover changes, the allometric equations of forest carbon mass also contribute to the simulated forest biomass uncertainty in ESMs. For example, the simulations from the HadGEM2 ESMs indiscriminately allocate exactly the same carbon mass in roots and in leaves (see Supplementary Table S2), which induces uncertainty in forest biomass estimates in these two simulations. Better representations of the dynamic carbon allocation schemes apportioning the carbon mass in leaves and roots should reduce the uncertainty of forest biomass estimates in ESMs^13,35^. Furthermore, all simulations except those from the IPSL ESMs consistently allocate too much carbon mass to wood but too little to leaves and roots. Although the proportions of carbon mass in wood and in roots simulated by the IPSL ESMs are within the observational uncertainty (Fig. 6c,d), the HadGEM2 ESMs and the MIROC ESMs have better carbon allocations for combined wood and root biomass (Fig. 6a). This result, which has also been shown in Negrón-Juárez *et al*.^14^, suggests the HadGEM2 ESMs and the MIROC ESMs include the coarse roots in the wood pool, while the IPSL ESMs report the coarse roots biomass as a part of root biomass. Therefore, it is more appropriate to compare the combined biomass in wood and in roots when estimating the uncertainty between ESMs, for individual models and the aggregated observations. Overestimation of wood and total biomass may also be attributable to positive biases in net primary productivity, poor parameterization of fire frequency and intensity^36^, lack of insect and disease processes in ESMs^37^, simplified characterization of canopy structure, overestimation of recruitment, and underestimation of tree mortality. As terrestrial ecosystem models evolve to simulate vegetation dynamics and demography^38–41^, development of improved and explicit representations of stand-level processes–including recruitment, succession, growth, mortality, and various kinds of disturbance–must be informed by systematic evaluation and benchmarking^42–44^, using *in situ* and remotely sensed observational data for burned area^45^, forest inventories^46^, primary production^47^, plant traits^48^, productivity and carbon residence times^49^, disturbance and extreme events^50,51^, mortality rates^52^, and other key forest energy and carbon fluxes^53^ and functional responses^54,55^.

Examination of the spin-up effect suggests that the uncertainty introduced at the beginning of the simulation in ESMs plays an important role in influencing the forest biomass estimates. Such spin-up issues have also been reported in soil carbon storage in CMIP5 outputs^56^. BNU-ESM and the IPSL ESMs simulate almost a unity *SR* value, demonstrating that the initial vegetation conditions from the spin-up processes in these models have substantial impact on present forest biomass estimates. As discussed above, the DGVM, land cover changes, and better representations and parameterizations of productivity, respiration and carbon turnover processes in the land models should be considered to improve model performance. Recent studies have shown that, in addition to mismatches in modeled compared to observed net primary production, carbon turnover processes are a major source of uncertainty in global vegetation models^4,57^. Simplified and not adequately represented turnover processes are contributing significantly to unrealistic magnitudes and spatial patterns of simulated biomass and to uncertainty in the response of vegetation carbon stocks to climate change^4,57^.

However, in addition to the uncertainties in the models, the observation-based product exhibits uncertainties as well. Sources of these uncertainties in the observation-based biomass estimate include the sensitivity of the radar signal to properties other than vegetation structure, the influence of non-forest vegetation on the signal, uncertainties in additional datasets (e.g. allometric databases, land cover, etc.) used for conversion of satellite measurements to biomass estimates, and the necromass component of vegetation in the observed growing stock volume. The application of allometric relationships derived on a leaf type level from a biomass compartment database has introduced relatively high uncertainties in observation-based estimates of leaf and root biomass compared to a moderate uncertainty in wood biomass. The uncertainty of the observation-based total biomass, however, is considered to be a conservative estimate and independent evaluations using upscaled forest inventory data have demonstrated the validity of the applied product at regional scales^24,58,59^.

Since the uncertainty of extratropical forest biomass estimates in ESMs can be from more factors in addition to the causes mentioned in this study, we suggest that the community utilize software packages to evaluate the performance of coupled ESMs whenever there is an improvement or modification in models. These tools can benefit the community by informing future development of ESMs. For instance, the International Land Model Benchmarking (ILAMB) project^44^ provides a means to not only provide the statistics of model outputs but also quickly and easily evaluate how model performance changes with each modification in ESMs through skill score spreads. Because of uncertainties in observational data, ILAMB incorporated multiple observational data sets for the same variable to provide the user with some indication of that observational uncertainty. With an increasing or a decreasing score, researchers are able to determine whether a modification in ESMs will reduce the model uncertainty in forest biomass estimates.

## Conclusion

We evaluated forest biomass from eight CMIP5 ESMs using a biomass dataset for the northern extratropical latitudes that was synthesized from radar remote sensing of stem volume and ground-based observations of wood density and allometric relationships between biomass compartments. ESMs exhibited large biases in forest distribution, forest fraction, and carbon mass that contributed to the overall uncertainty in forest total biomass. We showed that forest total biomass is positively correlated with precipitation variations with temperature becoming equally important at higher latitudes. Finally, we showed that uncertainties in the pre-industrial forest biomass (spin-up) persisted into the contemporary period in most ESMs. To improve future analysis of vegetation biomass, we recommend saving and archiving PFT-level carbon stocks and fluxes, which can be used for analysis and benchmarking. Routine evaluation of model biomass in all compartments and of the spatial distribution of vegetation types is important for identifying sources of uncertainty in carbon cycle predictions.

## Methods

### Model outputs

Outputs of eight Earth system model (ESM) simulations from the CMIP5 archive with the historical experiment “r1i1p1” (see Supplementary Table S1) are selected based on the data availability of carbon density of individual plant functional types (PFTs) for all biomass compartments including leaf, wood, root, and the total biomass. The model outputs contain climatic variables as well as detailed information about forest biomass for various PFTs (see Supplementary Table S4). Two climatic variables, the precipitation rate (PR) and the surface temperature (TAS), are analyzed to investigate the spatial correlations of forest biomass and climate. The forest biomass in CMIP5 archive stores a single carbon density derived from the area-weighted carbon density of all vegetation types in each grid cell. Hence, forests and grasses have the same carbon density value as long as their area fractions are non-zero within a grid cell. In this study, forest carbon density (kg C m^−2^) is converted into carbon mass (petagrams of carbon, Pg C). Data during 1982–2005 is averaged to represent the climatological mean status during the contemporary period, while that during the pre-industrial period is based on the climatological mean status during 1861–1885.

### Observation-based data sets

The applied observation-based forest biomass product^24^ (available from http://www.bgc-jena.mpg.de/geodb/projects/Home.php) provides vegetation carbon density and its uncertainty covering northern boreal and temperate forests (30°N–80°N) at 0.01° resolution. First, forest biomass in stems was derived from a radar remote sensing growing stock volume (GSV) product^58,59^, which integrates observations between October 2009 and February 2011, and information on wood density. Subsequently, the other biomass compartments (branches, roots, foliage) have been estimated based on a database of allometric relationships to stems. Furthermore, precipitation flux and surface temperature from the Global Soil Wetness Project Phase 3 (GSWP3) forcing data products (0.5° × 0.5°)^60^ serve as the observational climatic data. For land cover types, we adopt the land cover definitions from 1 km × 1 km global land cover classification for the year 2000 (GLC2000) database^31^ (see Supplementary Table S4). We reproject GLC2000 (applying nearest-neighbor interpolation) in order to match the resolution of the observation-based biomass product. To compare to each ESM’s output, this product is further upscaled to eight data sets with coarser grid resolutions that are the same as the eight selected CMIP5 model simulations (see Supplementary Table S1) while retaining the PFT-level biomass distributions (i.e., different carbon densities instead of a single area-weighted carbon density for each PFT within a grid cell).

### Common–grid land mask

We verify the data availability at each grid cell for the upscaled observational data sets and the ESM output. A grid cell is masked out when either observational or modeled forest biomass is unavailable at that grid cell. Due to different PFT distributions and grid resolutions, the available grid cells vary in each ESM. The total carbon stock estimated from the upscaled observational data sets, even though they are upscaled from the same product, are hence different. Therefore, we use relative errors, in addition to the total carbon stock, to evaluate the performance of each ESM output.

### Land cover types and forest fractions

The forest fractions for observation-based data are determined by GLC2000. The GLC2000 data is upscaled to coarser resolutions, varying from 1.25° × 1.875° to 2.8125° × 2.8125° (see Supplementary Table S1), according to each model’s setup. We classify four major forest types including broadleaf evergreen trees, needleleaf evergreen trees, broadleaf deciduous trees, and needleleaf deciduous trees (see Supplementary Table S5) when evaluating modeled forest distributions in Whittaker climate space. Alternatively, we combine the first ten classes in GLC2000 as the “lumped” forest type^24^ to quantify the uncertainty caused by spin-up processes in ESMs (see Supplementary Table S6). For each ESM output, modeled forest fractions are compared to that from those upscaled GLC2000 data at the same grid resolution. Uncertainty due to different forest definitions between GLC2000 and ESMs can be minimized by applying a common-grid land mask with various forest fraction thresholds (*F*_*f*_). The common-grid land mask keeps only the grid cells where both model outputs and observations have non-zero forest fractions as well as valid biomass values of forest types. *F*_*f*_ ranges from 0 to 0.9 with an increment of 0.1. Grid cells with forest fractions lower than or equal to *F*_*f*_ are masked out in both model outputs and observations. A higher *F*_*f*_ means that the carbon pool in grid cells composed of a larger proportion of forest-only biomass, i.e., the uncertainty caused by assigning a fixed carbon density for all PFTs in the ESMs is reduced.

### Spatial correlations and skill scores

Spatial differences between model outputs and observations are compared in terms of forest fractions and forest total carbon mass. We apply a 11 × 11 moving window to compute the spatial correlation between forest total carbon mass and precipitation rate as well as that between forest total carbon mass and surface temperature at the default forest fraction threshold (*F*_*f*_ = 0) over extratropical regions in the Northern Hemisphere. The spread of biomass in the precipitation–temperature space is lumped into two (broadleaf and needleleaf trees) and four categories (broadleaf evergreen, needleleaf evergreen, broadleaf deciduous, and needleleaf deciduous trees) depending on the PFT definitions in each model. A Taylor diagram^61^ is also produced to assess the spatial uncertainty due to *F*_*f*_ through the correlation, the root-mean-square difference and the ratio of the variances of forest total carbon mass from observations and from model outputs. Due to different spatial resolutions in each model, we use standardized deviations from each model’s variance normalized by the variance from the observations upscaled to its resolution. Model performance is evaluated through the skill scores (*S*)^61^:

1 $${S}_{i}=\frac{{\rm{4}}\times (1+{R}_{i})}{(\frac{{\sigma }_{i}}{{\sigma }_{r}}+\frac{{\sigma }_{r}}{{\sigma }_{i}})({\rm{1}}+{R}_{0})}$$

where *R*_*i*_ is the spatial pattern correlation of forest total carbon mass between model *i* and the upscaled observation at its resolution, *σ*_*i*_ the variance of modeled forest total carbon mass, *σ*_*r*_ the variance of observed forest total carbon mass. We compute ten different *R*_*i*_ values for each model *i* by varying *F*_*f*_ from 0 to 0.9 with an increment of 0.1. The maximum value of *R*_*i*_ among the ten *F*_*f*_ cases is assigned to *R*_0_.

### Relative error

To quantify the modeled forest biomass uncertainty, we measure the relative error (*ER*) with the common-grid land mask applied to evaluate the discrepancies of the magnitude of forest carbon amount between models and that from upscaled observational data:

2 $$ER=\frac{\sum _{i=1}^{n}{y}_{i}-\sum _{i=1}^{n}{x}_{i}}{\sum _{i=1}^{n}{x}_{i}}$$

where *n* is the total available grid cells, *y*_*i*_ the global forest total biomass from model outputs and *x*_*i*_ the global forest total biomass upscaled from the observational MPI–BGIv3 data set at grid cell *i*. The magnitude of observed global forest total biomass depends on data availability in each model output with the common-grid land mask applied. The multi-model average of forest total biomass is calculated based on the average of modeled outputs and upscaled observations for all ESMs using Eq. (2). In addition to analyzing the grid area-weighted results, we further examine the forest biomass with detailed carbon mass in individual PFTs at each grid cell from the HadGEM2-ES outputs. Grid cell-level uncertainty is computed by averaging *ER* over *n* available grid cells at each grid cell *i*:

3 $$E{R}_{grid}=\frac{1}{n}\,\sum _{i=1}^{n}({y}_{i}-{x}_{i})$$

In the present study, we evaluate *ER* for forest total biomass (*ER*_*total*_) as well as biomass in leaves (*ER*_*leaf*_), wood (*ER*_*wood*_), and roots (*ER*_*root*_). Because of the inconsistent definition of roots in ESMs, we also compute *ER* for the combined wood and roots biomass (*ER*_*wood*+*root*_). A model has less uncertainty in forest carbon mass estimates when *ER* approaches zero.

### Spin-up effects

The impact of the initial-to-equilibrium state from spin-up inputs on simulated forest biomass can be evaluated by the ratios of *ER*_*grid*_ values during the PI period and the modern time, i.e.,

4 $$SR=\frac{E{R}_{grid,PI}}{E{R}_{grid,contemporary}}$$

Note that we utilize the observations in contemporary years as the reference values for both *ER*_*grid*,*PI*_ and *ER*_*grid*,*contemporary*_ due to the absence of forest biomass information during the PI period. Nevertheless, it is suggested that higher possibilities of uncertainty in future carbon pool projections may be influenced by the inputs from spin-up results when *SR* values approach unity.

## Electronic supplementary material

Supplementary Information
