# Supplementary material for: Uncertainty Quantification of Extratropical Forest Biomass in CMIP5 Models over the Northern Hemisphere
Source: Sci Rep. 2018 Jul 19;8:10962. doi: 10.1038/s41598-018-29227-7 (PMC6053416; doi:10.1038/s41598-018-29227-7)
Supplement: Supplementary file 1 — Supplementary Information [file 41598_2018_29227_MOESM1_ESM.pdf]

Supplementary Information for

Uncertainty Quantification of Extratropical Forest Biomass in CMIP5 Models over the Northern Hemisphere

Cheng-En Yang, Jiafu Mao, Forrest M. Hoffman, Daniel M. Ricciuto, Joshua S. Fu, Chris D. Jones, and Martin Thurner

Table S1. Summary of eight CMIP5 earth system models used in this study.

Modeling center	Model	Institution	Resolution*	Grid cells	Land carbon cycle component	Dynamic vegetation	Land cover change	Reference
GCESS	BNU-ESM	College of Global Change and Earth System Science, Beijing Normal University	2.8125° × 2.8125°	64 × 128	CoLM3 and BNU-DGVM	Yes	Yes†	1–3
MOHC	HadGEM2-CC<br>HadGEM2-ES	Met Office Hadley Centre (additional HadGEM2-ES realizations contributed by Instituto Nacional de Pesquisas Espaciais)	1.25° × 1.875°	145 × 192	JULES and TRIFFID	Yes	Yes	4–9
IPSL	IPSL-CM5A-LR<br>IPSL-CM5A-MR<br>IPSL-CM5B-LR	Institut Pierre-Simon Laplace	1.9° × 3.75°<br>1.25° × 2.5°<br>1.9° × 3.75°	96 × 96<br>143 × 144<br>96 × 96	ORCHIDEE	No	Yes‡	10, 11
MIROC	MIROC-ESM<br>MIROC-ESM-CHEM	Japan Agency for Marine-Earth Science and Technology, Atmosphere and Ocean Research Institute (The University of Tokyo), and National Institute for Environmental Studies	2.8125° × 2.8125°	64 × 128	MATSIRO and SEIB–DGVM	Yes	Yes	12, 13

* latitude × longitude.
† no land cover change due to anthropogenic land use change.
‡ no prognostic land cover change.

Table S2. Global total carbon mass (Pg C) of forest compartments across 30°N–80°N for CMIP5 models (*Model*) and upscaled observations (*Obs.*) at $F_f = 0$ during the contemporary period (Modern) and the pre-industrial (PI) periods. "Wood+Root" is the sum of biomass in wood and in roots. Institutes include (A) GCESS, (B) MOHC, (C) IPSL, and (D) MIROC. Model outputs are from (1) BNU-ESM, (2) HadGEM2-CC, (3) HadGEM2-ES, (3_{PFT}) HadGEM2-ES with detailed grid-level biomass for each PFT, (4) IPSL-CM5A-LR, (5) IPSL-CM5A-MR, (6) IPSL-CM5B-LR, (7) MIROC-ESM, and (8) MIROC-ESM-CHEM. Each modeling center (B), (C), and (D) has "Mean" values from the averages of all available model outputs within a center. The "Multi-Model Mean" values are the averages of all model outputs ($n = 9$) whereas the "Institutional Mean" are the averages of the four modeling centers' mean values ($n = 4$). Note that in each model resolution a common grid mask is applied to both *Model* and *Obs.*

**Modeling center**	**Model**	**Type**	**Total**		**Leaf**		**Wood**		**Root**		**Wood+Root**	
			Modern	PI	Modern	PI	Modern	PI	Modern	PI	Modern	PI
(A)	(1)	*Obs.*	37.35	—	1.81	—	27.23	—	8.30	—	35.53	—
		Model	150.59	141.20	1.57	1.37	147.21	138.26	1.81	1.57	149.02	139.83
(B)	(2)	*Obs.*	71.36	—	3.70	—	51.93	—	15.73	—	67.66	—
		Model	51.11	68.15	2.44	3.31	46.23	61.52	2.44	3.31	48.67	64.83
	(3)	*Obs.*	71.36	—	3.70	—	51.93	—	15.73	—	67.66	—
		Model	58.01	74.16	2.74	3.61	52.54	66.95	2.74	3.61	55.27	70.55
	(3_{PFT})	*Obs.*	71.36	—	3.70	—	51.93	—	15.73	—	67.66	—
		Model	81.81	—	3.62	—	74.57	—	3.62	—	78.19	—
	Mean	*Obs.*	71.36	—	3.70	—	51.93	—	15.73	—	67.66	—
		Model	63.64	71.16	2.93	3.46	57.78	64.24	2.93	3.46	60.71	67.69
(C)	(4)	*Obs.*	70.24	—	3.65	—	51.10	—	15.49	—	66.59	—
		Model	189.86	201.53	4.02	4.16	143.44	152.36	38.48	40.73	181.91	193.09
	(5)	*Obs.*	69.11	—	3.59	—	50.24	—	15.27	—	65.51	—
		Model	203.80	213.54	4.00	4.16	154.83	162.20	41.10	42.97	195.93	205.16
	(6)	*Obs.*	70.24	—	3.65	—	51.10	—	15.49	—	66.59	—
		Model	138.26	140.91	3.73	3.55	102.66	104.76	28.16	28.65	130.82	133.41
	Mean	*Obs.*	69.86	—	3.63	—	50.81	—	15.42	—	66.23	—
		Model	177.31	185.33	3.92	3.96	133.64	139.77	35.91	37.45	169.55	177.22
(D)	(7)	*Obs.*	66.70	—	3.47	—	48.53	—	14.69	—	63.22	—
		Model	78.73	110.38	3.59	4.51	67.73	96.51	2.85	3.64	70.58	100.14
	(8)	*Obs.*	66.33	—	3.46	—	48.28	—	14.60	—	62.88	—
		Model	71.64	118.55	3.19	4.77	61.78	104.06	2.58	3.77	64.36	107.83
	Mean	*Obs.*	66.52	—	3.47	—	48.41	—	14.65	—	63.05	—
		Model	75.19	114.47	3.39	4.64	64.76	100.29	2.72	3.71	67.47	103.99
Multi-Model Mean		*Obs.*	66.01	—	3.41	—	50.55	—	13.21	—	63.76	—
($n = 9$)		*Model*	113.76	133.55	3.21	3.68	92.04	110.83	15.10	16.03	107.14	126.86
Institutional Mean		*Obs.*	61.27	—	3.15	—	44.59	—	13.52	—	58.12	—
($n = 4$)		*Model*	116.68	128.04	2.95	3.36	100.85	110.64	10.84	11.55	111.69	122.18

Table S3. Grid cell-level relative errors of all compartments ($ER_{grid,total}$), leaves ($ER_{grid,leaf}$), wood ($ER_{grid,wood}$), roots ($ER_{grid,root}$), and wood plus roots ($ER_{grid,wood+root}$) for each model during the contemporary period at $F_f = 0$. Numbers represent global mean and one standard deviation of ER_grid values for all available grid cells after masks applied. Model numbers are the same as shown in Supplementary Table S2.

Model	$ER_{grid,total}$	$ER_{grid,leaf}$	$ER_{grid,wood}$	$ER_{grid,root}$	$ER_{grid,wood+root}$
(1)	168.0 ± 1702.5	20.6 ± 202.6	265.3 ± 2811.7	10.1 ± 151.4	182.7 ± 1853.9
(2)	61.7 ± 1665.8	22.5 ± 332.5	99.1 ± 2839.9	12.5 ± 296.6	74.1 ± 2093.3
(3)	63.8 ± 1792.9	21.3 ± 339.5	103.3 ± 3061.8	12.3 ± 307.5	77.0 ± 2253.7
(4)	281.0 ± 2876.4	55.4 ± 502.1	327.3 ± 3628.5	207.7 ± 2116.5	287.3 ± 3126.4
(5)	208.3 ± 2009.3	32.8 ± 284.8	252.7 ± 2551.8	156.3 ± 1535.6	217.2 ± 2115.1
(6)	209.4 ± 2234.3	53.2 ± 480.4	225.9 ± 2722.1	152.3 ± 1622.1	200.9 ± 2355.7
(7)	91.1 ± 893.0	51.2 ± 574.4	116.7 ± 1206.0	14.9 ± 167.0	83.0 ± 841.8
(8)	85.2 ± 886.0	51.7 ± 531.8	101.4 ± 1114.7	15.6 ± 168.6	75.9 ± 844.5

Table S4. Global land cover classification for the year 2000 from GLC2000 and CMIP5 models. Institutions (A)-(D) are the same as shown in Supplementary Table S2.

Class	GLC2000	(A)	(B)	(C)	(D)
1	Tree Cover, broadleaf, evergreen	Needleleaf evergreen temperate tree	Broadleaf trees	Bare soil	Tropical forest
2	Tree Cover, broadleaf, deciduous, closed	Needleleaf evergreen boreal tree	Needleleaf trees	Tropical broadleaf evergreen trees	Temperate evergreen
3	Tree Cover, broadleaf, deciduous, open	Needleleaf deciduous boreal tree	C3 (temperate) grass	Tropical broadleaf raingreen trees	Temperate deciduous
4	Tree Cover, needleleaf, evergreen	Broadleaf evergreen tropical tree	C4 (tropical) grass	Temperate needleleaf evergreen trees	Boreal evergreen
5	Tree Cover, needleleaf, deciduous	Broadleaf evergreen temperate tree	Shrubs	Temperate broadleaf evergreen trees	Boreal deciduous
6	Tree Cover, mixed leaf type	Broadleaf deciduous tropical tree	Urban	Temperate broadleaf summergreen trees	C3 grass
7	Tree Cover, regularly flooded, fresh water	Broadleaf deciduous temperate tree	Inland water	Boreal needleleaf evergreen trees	C4 grass
8	Tree Cover, regularly flooded, saline water	Broadleaf deciduous boreal tree	Bare soil	Boreal broadleaf summergreen trees	Crop
9	Mosaic: Tree cover / Other natural vegetation	Broadleaf evergreen shrub	Ice	Boreal needleleaf summergreen trees	Pasture
10	Tree Cover, burnt	Broadleaf deciduous temperate shrub		Natural C3 grass	Bare ground
11	Shrub Cover, closed-open, evergreen	Broadleaf deciduous boreal shrub		Natural C4 grass	Residual
12	Shrub Cover, closed-open, deciduous	C3 arctic grass		Agricultural C3 grass	
13	Herbaceous Cover, closed-open	C3 non-arctic grass		Agricultural C4 grass	
14	Sparse Herbaceous or sparse Shrub Cover	C4 grass			
15	Regularly flooded Shrub and/or Herbaceous Cover				

Table S4. (*continued*) Global land cover classification for the year 2000 from GLC2000 and CMIP5 models. Institutions (A)-(D) are the same as shown in Supplementary Table S2.

Class	GLC2000	(A)	(B)	(C)	(D)
16	Cultivated and managed areas				
17	Mosaic: Cropland / Tree Cover / Other natural vegetation				
18	Mosaic: Cropland / Shrub or Grass Cover				
19	Bare areas				
20	Water Bodies				
21	Snow and Ice				
22	Artificial surfaces and associated areas				

Table S5. Forest type definitions for Whittaker diagrams. Institutions (A)-(D) are the same as shown in Supplementary Table S2. Numbers represent the PFT classes defined in Supplementary Table S4. Forest types include broadleaf evergreen trees (BET), needleleaf evergreen trees (NET), broadleaf deciduous trees (BDT), and needleleaf deciduous trees (NDT). Note that MOHC models only have broadleaf trees (BT) and deciduous trees (DT).

Dataset	BET	NET	BDT	NDT	BT	NT
**GLC2000**	1	4	2, 3	5	1, 2, 3	4, 5
**(A)**	4, 5	1, 2	6, 7, 8	3	—	—
**(B)**	—	—	—	—	1	2
**(C)**	2, 5	4, 7	3, 6, 8	9	—	—
**(D)**	1	2, 4	3	5	—	—

Table S6. Definitions of forest types in GLC2000 and each model. Numbers represent the classes defined in Supplementary Table S4. Unavailable land types are denoted by N/A. The GLC2000 forest definition is adopted from Thurner *et al.* (2014)[14].

Forest Type	GLC2000	(A)	(B)	(C)	(D)
**Broadleaf**	1, 2, 3	5, 7, 8	1	5, 6, 8	N/A
**Needleleaf**	4, 5	1, 2, 3	2	4, 7, 9	N/A
**Evergreen**	1, 4	1, 2, 5	N/A	4, 5, 7	2, 4
**Deciduous**	2, 3, 5	3, 7, 8	N/A	6, 8, 9	3, 5
**Lumped**	1 – 10	1 – 3, 5, 7, 8	1, 2	4 – 9	2 – 5

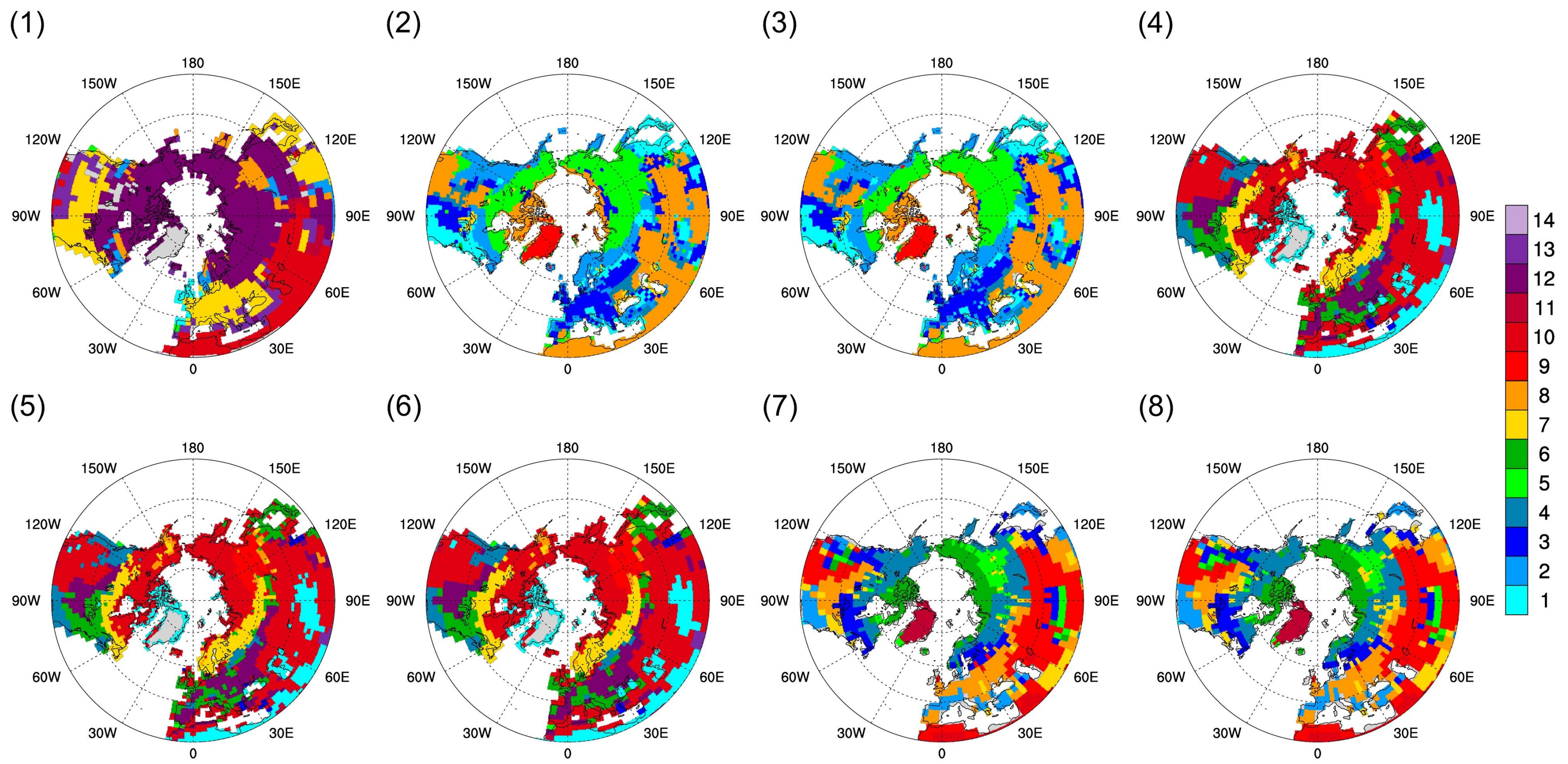

Figure S1. Dominant PFT in each model. PFT types are listed in Supplementary Table S4. Models include (1) BNU-ESM, (2) HadGEM2-CC, (3) HadGEM2-ES, (4) IPSL-CM5A-LR, (5) IPSL-CM5A-MR, (6) IPSL-CM5B-LR, (7) MIROC-ESM, and (8) MIROC-ESM-CHEM. [Maps were made using the NCAR Command Language v6.4.0 software, *http://dx.doi.org/10.5065/D6WD3XH5*.]

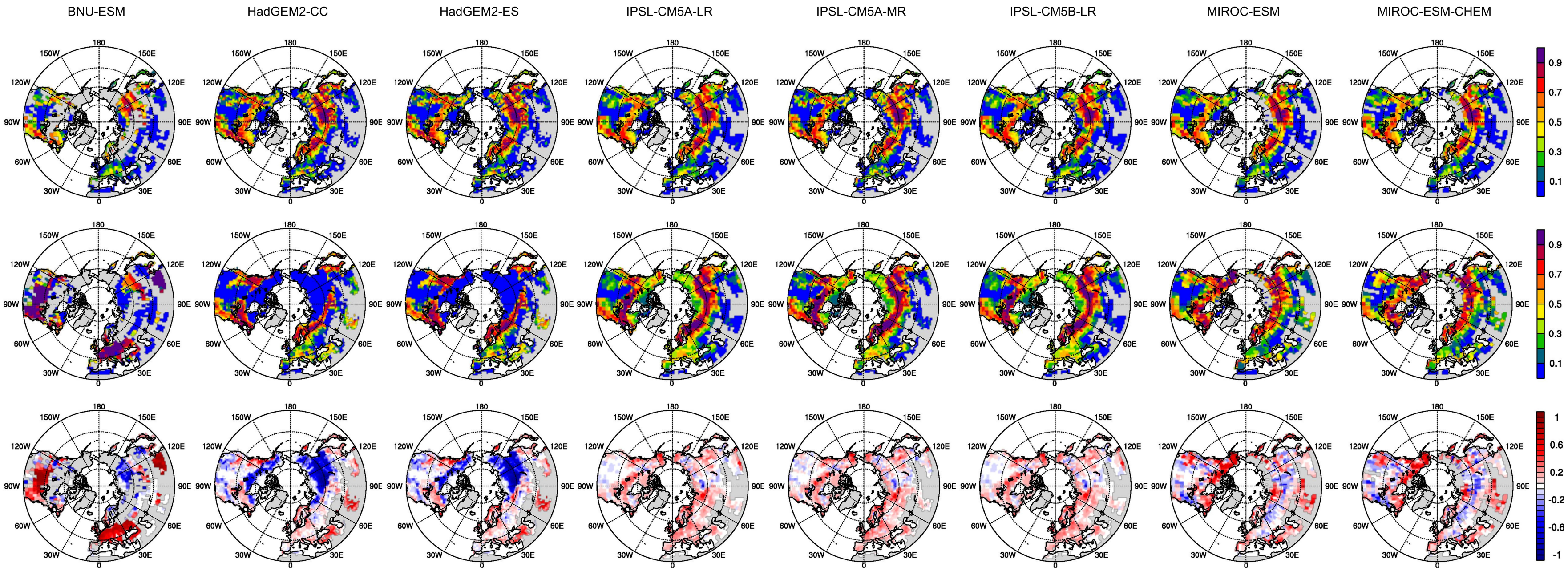

Figure S2. Same as Fig. 1 except for all ESMs (column-wise, left to right): BNU-ESM, HadGEM2-CC, HadGEM2-ES, IPSL-CM5A-LR, IPSL-CM5A-MR, IPSL-CM5B-LR, MIROC-ESM, and MIROC-ESM-CHEM. [Maps were made using the NCAR Command Language v6.4.0 software, *http://dx.doi.org/10.5065/D6WD3XH5*.]

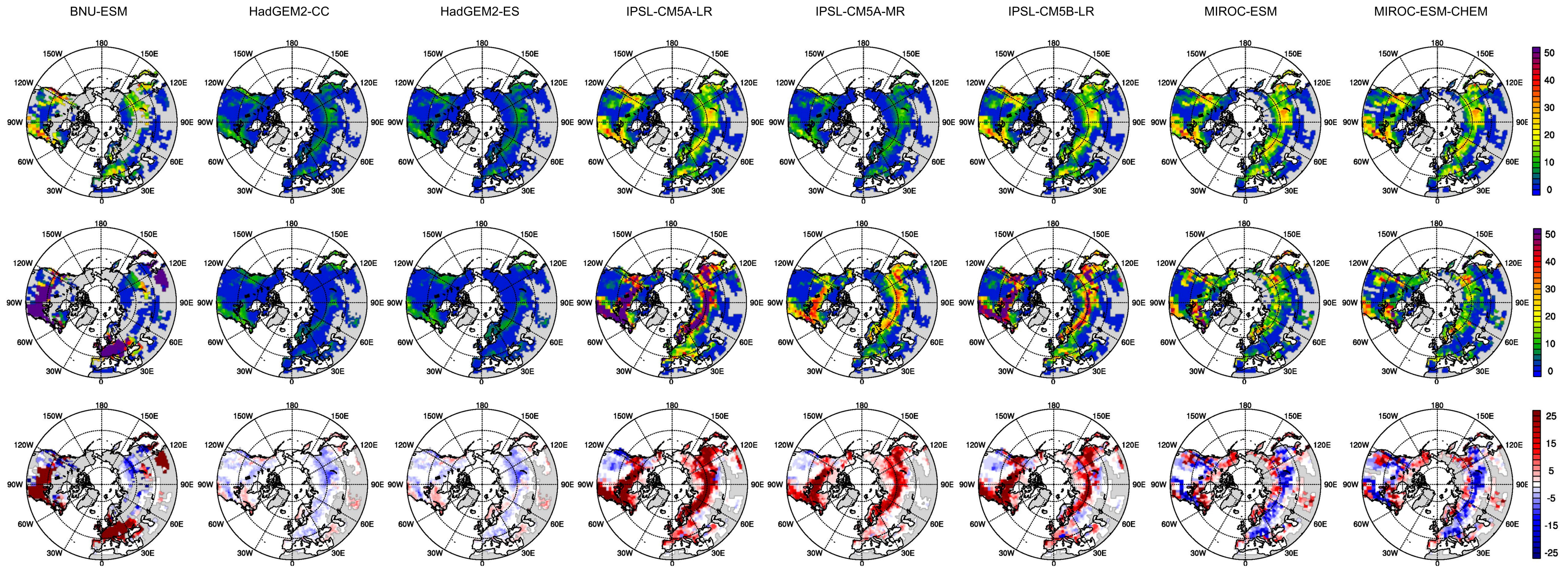

Figure S3. Same as Fig. 2 except for all ESMs (column-wise, left to right): BNU-ESM, HadGEM2-CC, HadGEM2-ES, IPSL-CM5A-LR, IPSL-CM5A-MR, IPSL-CM5B-LR, MIROC-ESM, and MIROC-ESM-CHEM. [Maps were made using the NCAR Command Language v6.4.0 software, *http://dx.doi.org/10.5065/D6WD3XH5*.]

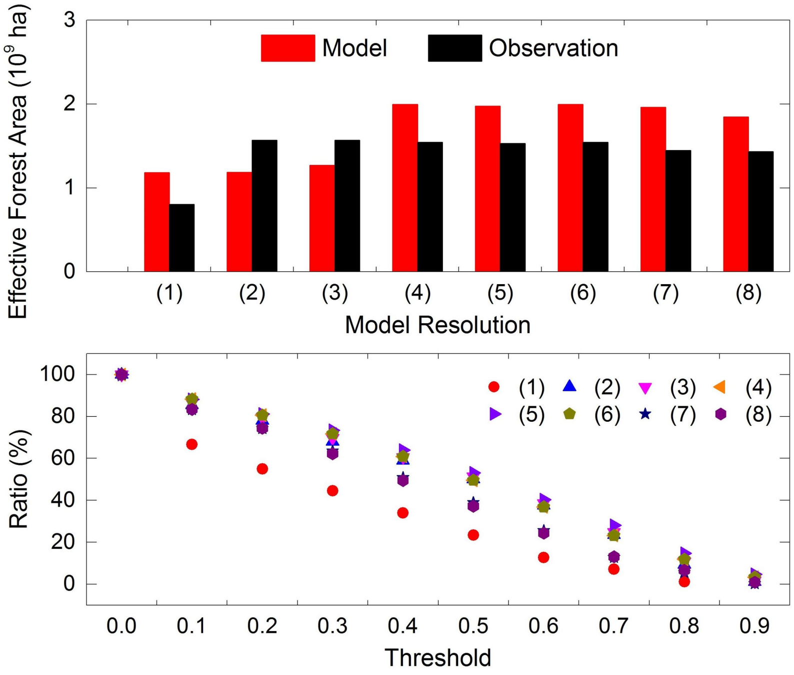

Figure S4. (Top) Effective forest area of forest total carbon mass at default forest threshold ($F_f = 0$) from model outputs (red) and from upscaled observations (black) during the contemporary period. A common-grid mask in both observed and modeled values is applied. (Bottom) The ratios of effective forest area at various forest fraction thresholds to that at $F_f = 0$. Models include (1) BNU-ESM, (2) HadGEM2-CC, (3) HadGEM2-ES, (4) IPSL-CM5A-LR, (5) IPSL-CM5A-MR, (6) IPSL-CM5B-LR, (7) MIROC-ESM, and (8) MIROC-ESM-CHEM. [The figure was made using the Origin software (OriginLab, Northampton, MA, USA).]

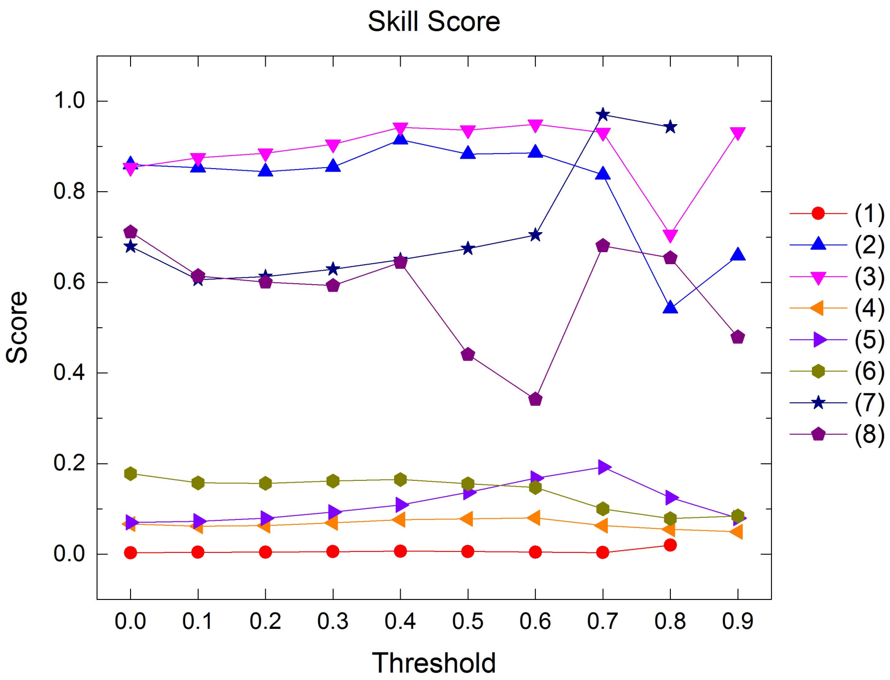

Figure S5. Skill scores for modeled global forest carbon mass in different forest fraction thresholds during the contemporary period. Models include (1) BNU-ESM (red), (2) HadGEM2-CC (green), (3) HadGEM2-ES (blue), (4) IPSL-CM5A-LR (magenta), (5) IPSL-CM5A-MR (light green), (6) IPSL-CM5B-LR (violet), (7) MIROC-ESM (light blue), and (8) MIROC-ESM-CHEM (dark brown). [The figure was made using the Origin software (OriginLab, Northampton, MA, USA).]

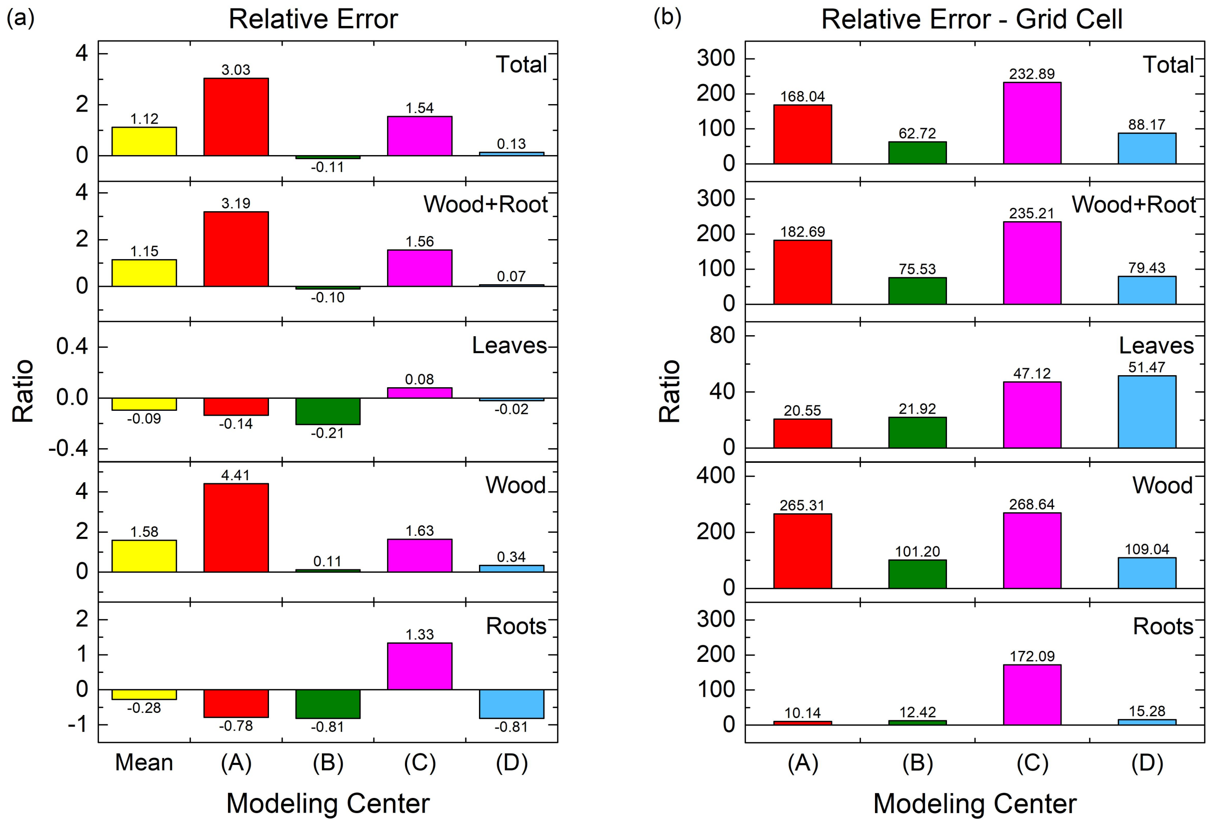

Figure S6. Institutional averaged (a) relative errors and (b) relative errors at grid cell level for individual forest compartments (top to bottom) during the contemporary period: all compartments, wood and roots, leaves, wood, and roots. Letters for modeling centers are the same as used in Supplementary Table S2. "Mean" represents the average of all four modeling centers' outputs. [The figure was made using the Origin software (OriginLab, Northampton, MA, USA).]

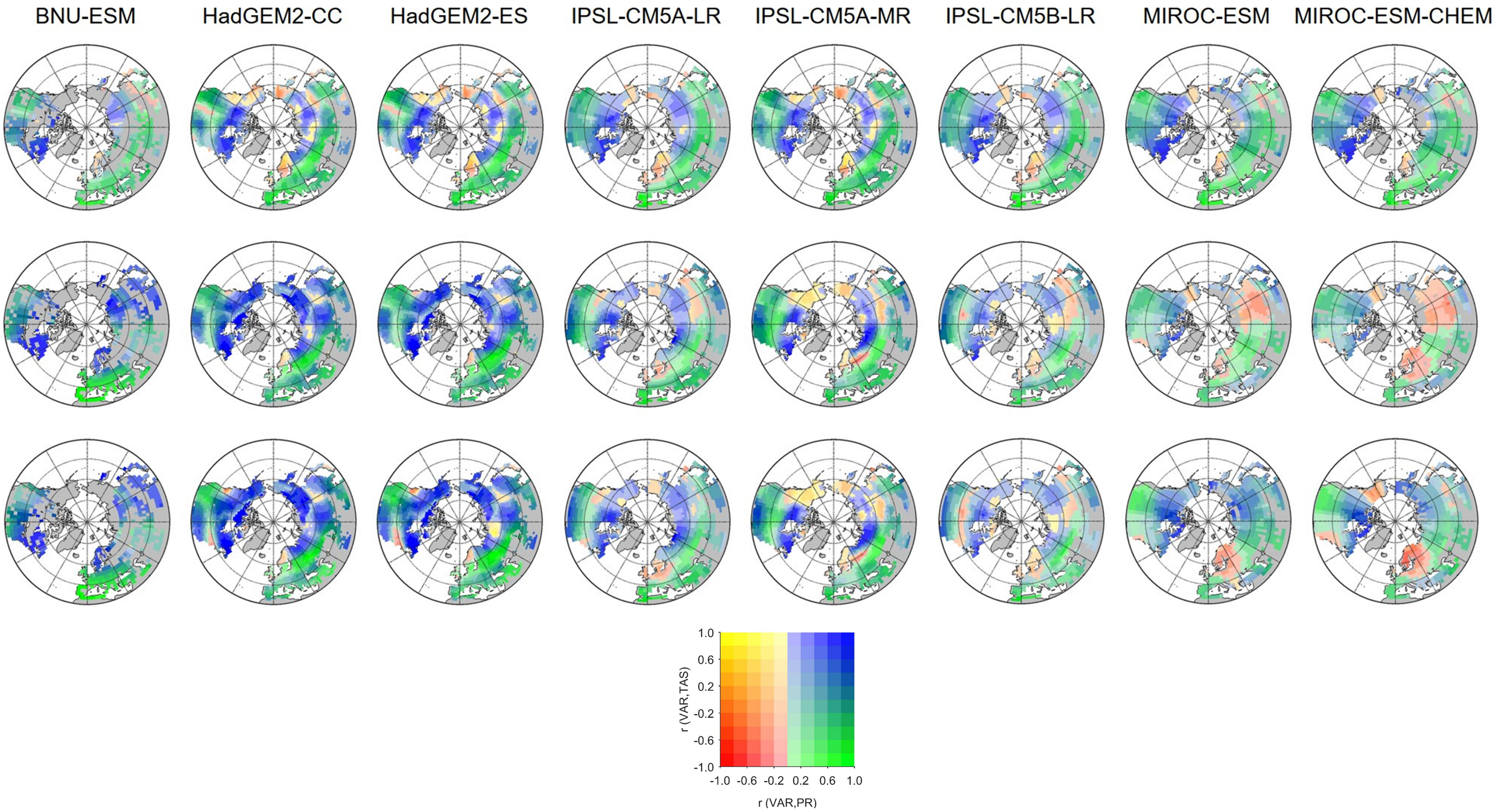

Figure S7. Same as Fig 7 except for all ESM resolutions (left to right, columnwise): BNU-ESM, HadGEM2-CC, HadGEM2-ES, IPSL-CM5A-LR, IPSL-CM5A-MR, IPSL-CM5B-LR, MIROC-ESM, and MIROC-ESM-CHEM. [Maps were made using Matlab version 2016a, *https://www.mathworks.com/products/matlab.html*.]

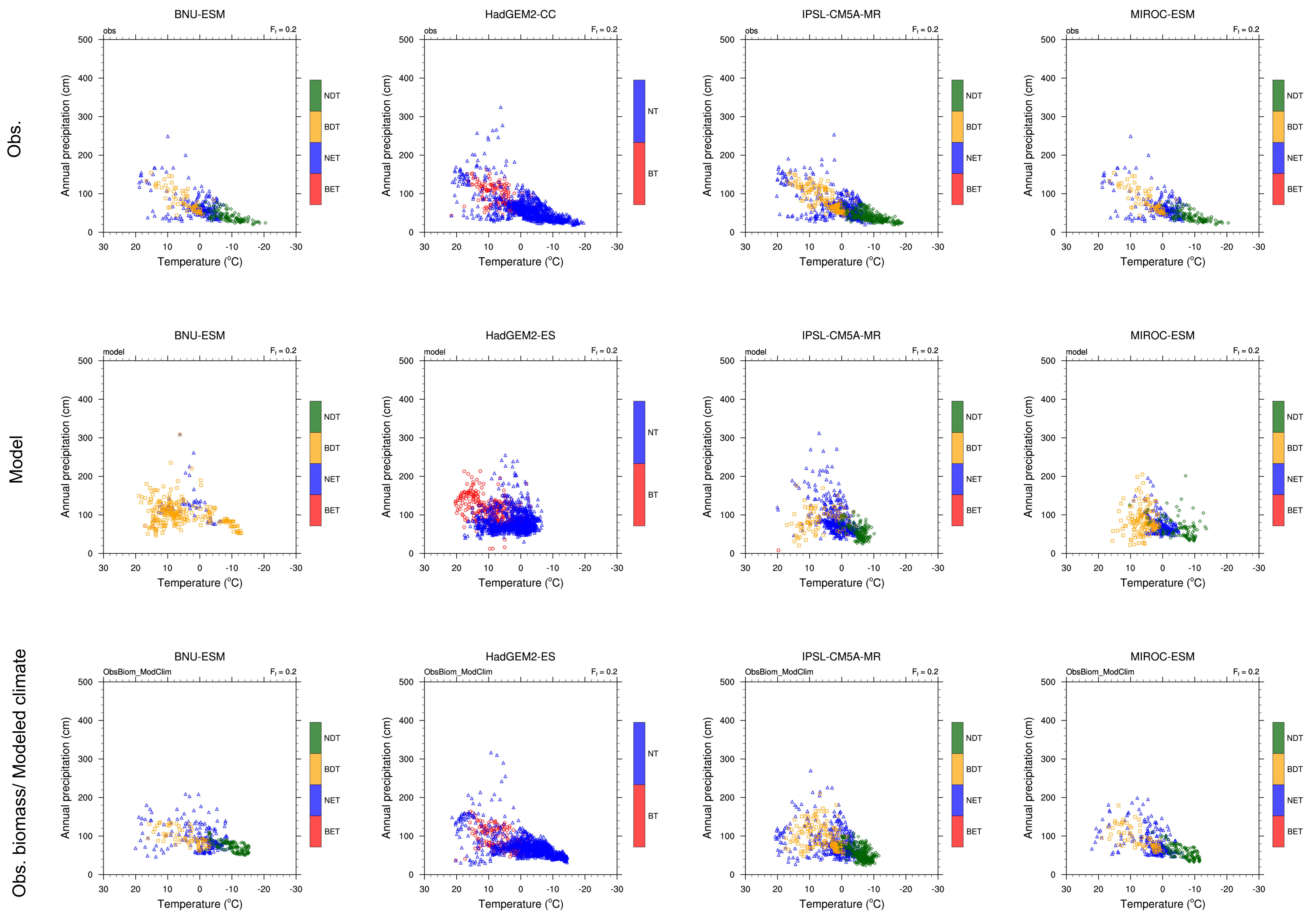

Figure S8. Whittaker diagrams of various forest types ($F_f = 0.2$) for upscaled observations (top row), models (center row), and observed biomass in modeled climate space for four ESMs (left to right) during the contemporary period: BNU-ESM, HadGEM2-ES, IPSL-CM5A-MR, and MIROC-ESM. Colors represent different PFT categories defined in Supplementary Table S5. [Maps were made using the NCAR Command Language v6.4.0 software, *http://dx.doi.org/10.5065/D6WD3XH5*.]

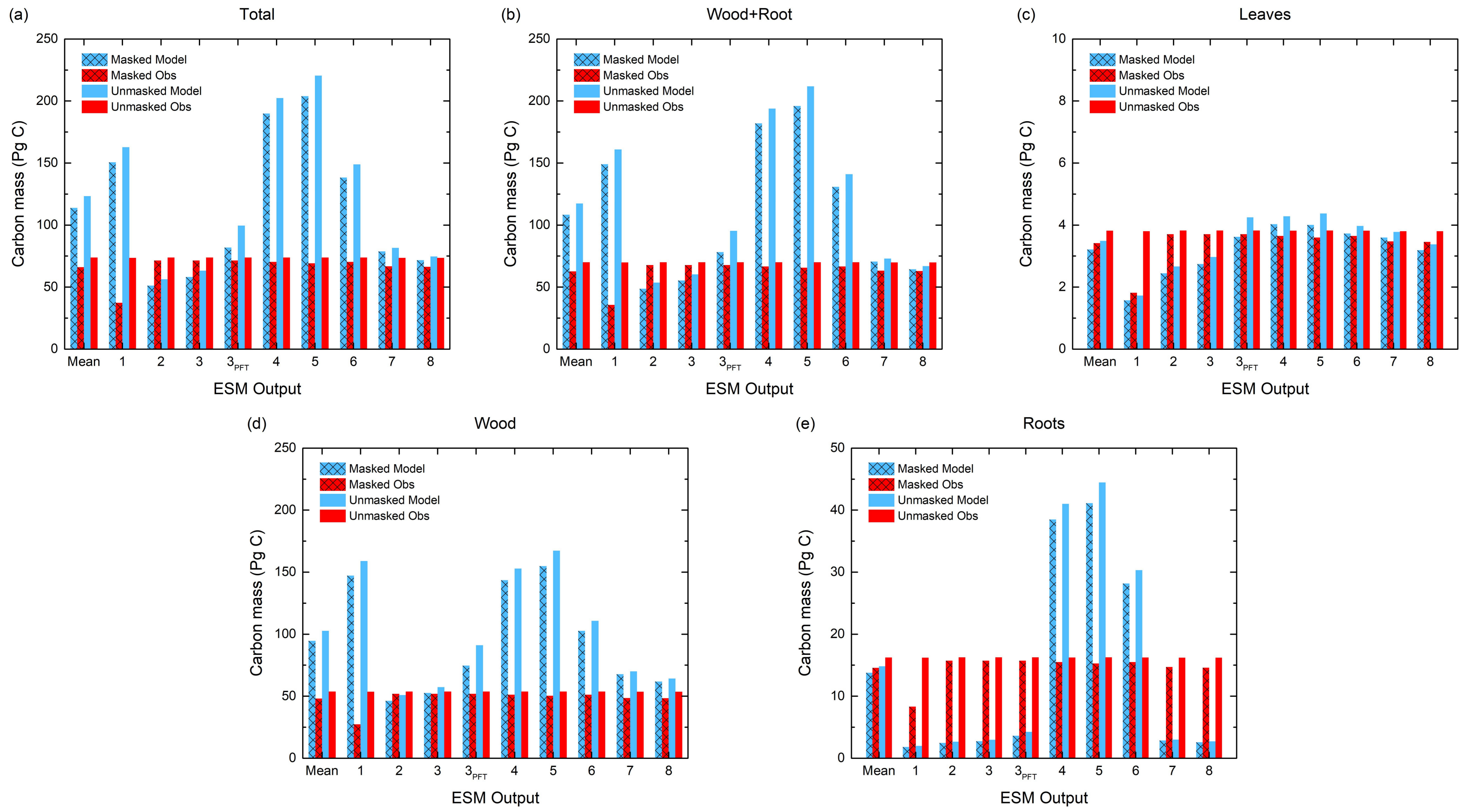

Figure S9. Mask and unmasked (checkered columns) forest total carbon mass (Pg C) for ESM outputs (blue) and upscaled observational data sets (red) in (a) total biomass, (b) wood and roots, (c) leaves, (d) wood, and (e) roots during the contemporary period. ESM numbers are the same as used in Supplementary Table S2. "Mean" represents the average of all ESM outputs. [The figure was made using the Origin software (OriginLab, Northampton, MA, USA).]

References

1. Dai, Y. *et al.* The common land model. *Bull. Amer. Meteor. Soc.* **84**, 1013–1023 (2003).
2. Dai, Y., Dickinson, R. E. & Wang, Y.-P. A two-big-leaf model for canopy temperature, photosynthesis, and stomatal conductance. *J. Clim.* **17**, 2281–2299 (2004).
3. Ji, D. *et al.* Description and basic evaluation of Beijing Normal University Earth System Model (BNU-ESM) version 1. *Geosci. Model Dev.* **7**, 2039–2064 (2014).
4. Collins, W. J. *et al.* Development and evaluation of an Earth-System model – HadGEM2. *Geosci. Model Dev.* **4**, 1051–1075 (2011).
5. Cox, P. M. *et al.* The impact of new land surface physics on the GCM simulation of climate and climate sensitivity. *Clim. Dynamics* **15**, 183–203 (1999).
6. Cox, P. M. Description of the TRIFFID Dynamic Global Vegetation Model. Tech. Rep. Hadley Centre Technical Note 24, Met Office, UK. (2001).
7. Essery, R. L. H., Best, M. J., Betts, R. A., Cox, P. M. & Taylor, C. M. Explicit Representation of Subgrid Heterogeneity in a GCM Land Surface Scheme. *J. Hydrometeor.* **4**, 530–543 (2003).
8. Jones, C. D. *et al.* The HadGEM2-ES implementation of CMIP5 centennial simulations. *Geosci. Model Dev.* **4**, 543–570 (2011).
9. Martin, G. M. *et al.* The HadGEM2 family of Met Office Unified Model climate configurations. *Geosci. Model Dev.* **4**, 723–757 (2011).
10. Dufresne, J.-L. *et al.* Climate change projections using the IPSL-CM5 Earth System Model: from CMIP3 to CMIP5. *Clim. Dynam.* **40**, 2123–2165 (2013).
11. Krinner, G. *et al.* A dynamic global vegetation model for studies of the coupled atmosphere-biosphere system. *Global Biogeochem. Cycles* **19**, GB1015 (2005).
12. Sato, H., Itoh, A. & Kohyama, T. SEIB–DGVM: A new Dynamic Global Vegetation Model using a spatially explicit individual-based approach. *Ecolog. Modelling* **200**, 279–307 (2007).
13. Watanabe, S. *et al.* MIROC-ESM 2010: model description and basic results of CMIP5-20c3m experiments. *Geosci. Model Dev.* **4**, 845–872 (2011).
14. Thurner, M. *et al.* Carbon stock and density of northern boreal and temperate forests. *Global Ecol. Biogeogr.* **23**, 297–310 (2014).
